# An Introduction to Biomolecular Graphics

**DOI:** 10.1371/journal.pcbi.1000918

**Published:** 2010-08-26

**Authors:** Cameron Mura, Colin M. McCrimmon, Jason Vertrees, Michael R. Sawaya

**Affiliations:** 1 Department of Chemistry, University of Virginia, Charlottesville, Virginia, United States of America; 2 Schrödinger LLC, New York, New York, United States of America; 3 Howard Hughes Medical Institute, University of California, Los Angeles, California, United States of America; Whitehead Institute, United States of America

## Introduction

Biological function arises from detailed molecular structure, making it difficult to overemphasize the role of structural visualization and biomolecular graphics in shaping our current understanding of the molecular nature of biological systems. Indeed, one need only compare the number of three-dimensional (3D) structures illustrated in the first (1990) and fourth (2010) editions of Voet & Voet's *Biochemistry* in order to appreciate the profound communicative value of molecular graphics in modern biosciences, ranging from medicine and physiology to drug design and computational biology. Faced with a deluge of structural genomics results over the past decade, the cliché about a picture being worth a thousand words is quite poignant: The information “content” of carefully constructed molecular graphics can be immense. Because computer-based molecular visualization (MolVis) is such an effective means for exploring and analyzing structural data, this guide introduces the science and art of biomolecular graphics, both in principle and as practiced in structural and computational biology.

This guide is built around a series of practical case-studies, emphasizing the creation of biomolecular graphics for publication figures and animations. Intended primarily for those embarking upon their first illustrations, intermediate-level examples are also provided in order to facilitate the transition from novice user to advanced practitioner. For enhanced pedagogical value, the exact methods used to create each figure are provided to the reader in the form of heavily annotated computer scripts. Because the PyMOL [1] software package was used to create these illustrations, all materials (images, animations, scripts, etc.) have been made freely available as a dedicated section of the PyMOL wiki site (http://pymolwiki.org/PLoS). Additional background material on MolVis, including a detailed review of the underlying principles (Box 1), is provided as supporting information (Text S1). Further information can be found in the recent treatment by Bottomley and Helmerhorst [2], and in several reviews covering either small-molecule [3] or macromolecular visualization [4]–[6].

**Box 1. MolVis Concepts and Terminology**

**Raster, vector:** Two different ways to structure images, either as combinations of simple geometric objects such as points, lines, curves (vector graphics), or as a discrete 2D array of colored pixels (raster/bitmap). Vector graphics are arbitrarily scalable, whereas the fixed array of pixels in a bitmap leads to graininess (“pixelization”) upon zooming-in of raster graphics; see Box S1 in Text S1 and ref. [2] for further information.

**Graphics primitives:** Low-level geometric entities that are readily described in mathematical terms (lines, spheres, tetrahedra, etc.), and from which any complex shape, such as protein surfaces, can be constructed via solid geometry. Scenes are built from primitives, along with associated lighting, shading, and texturing properties; thus, primitives are how a scene is discretized for computer representation and manipulation. As an example, increasing PyMOL's “sphere_quality” beyond the default value of 1 yields smoother spheres (more triangles), while decreasing to 0 exposes the individual triangular primitives used to render spheres.

**Scene geometry, matrices:** Several matrices are used to transform a molecular scene (atomic coordinate-based) into the image (pixel-based) shown on the actual 2D display. Along with all the primitives that represent molecular properties (atoms, bonds, surfaces, etc.), many other scene data must also be carried through these transforms, including materials, colors, lighting, shading, clipping, and depth (*z*) buffer data—in other words, all the attributes that define a scene. In being mapped onto the viewing plane, a scene can be rendered in either a perspective (skewed viewing matrix) or orthoscopic (orthonormal viewing matrix) projection mode; the PyMOL settings “orthoscopic” and “field_of_view” adjust this behavior, and the viewing matrix can be retrieved/modified via the “get_view” / “set_view” pair of commands.

**Clipping planes:** The boundaries of a scene define a rectangular pyramid, with an apex at the camera(/eye), and the faces defined by top/bottom and right/left pairs of planes. In addition, far/near *clipping planes* can be defined behind/in front of a region of interest in this rectangular pyramid. Clipping plane geometry and behavior is adjustable; for instance, the PyMOL command “clip slab, 20” sets the slab thickness to 20 Å.

**Ray tracing:** A method to render photorealistic images by simulating the path of light rays through a scene, incorporating effects such as light sources, opacity, textures, atmospheric fog, and shading models. Ray tracing is computationally expensive for complex scenes, and more “realistic” (higher resolution) images require a greater density of light rays per pixel of the final image.

**Keyframes:** Reference markers, either in time (animations) or in space (interpolations), that serve as the end-points that bracket an interpolation stage. For instance, in a sequence of frames consisting of structural snapshots *S_1_*→···→*S_2_* ······ *S_n_*, *S_1_* and *S_2_* define the first pair of keyframes. Linearly interpolating the gaps between *S_1_* and *S_2_* is essentially a form of data-smoothening. Most movie-making functionalities incorporate the keyframe concept.

**Anti-aliasing:** A feature/setting in most MolVis programs (“antialias” in PyMOL) that greatly improves image quality by diminishing the jagged distortions (“aliasing”) of curves and diagonal lines that compose the geometric primitives of a scene.

## Getting Started: Preliminaries and Software Tools

The most important question to ask at the outset of a graphics project is “Is the image necessary?” A figure is likely unnecessary if its main point is more easily conveyed by a brief sentence of prose. For instance, if a manuscript illustrates the new structure of protein X, which is found to have a uniform root mean square deviation (RMSD)<0.5 Å to protein Y, then it may be more effective to simply write “proteins X and Y are virtually identical, to within 0.5 Å RMSD at all residues” rather than to create a structural alignment graphic to show this. Conversely, also consider cases in which a large swath of text can be replaced by a single, well-designed figure; for instance, visual images are more effective than words in describing intricate details of a ligand-binding site, or geometric features of particularly complex protein•protein interfaces. Similarly, there are instances (toward the right of Figure S1) when conceptual schematics surpass coordinate-based representations; in such cases, much of the figure-creation effort may involve third-party editing software, such as Adobe Illustrator, rather than actual MolVis/rendering programs such as PyMOL.

Once a figure is deemed necessary, the next step is to specifically articulate its purpose. Two major purposes of all biomolecular graphics are (i) to enable visualization of structures too small to be seen by the naked eye, and (ii) to simplify the inherent complexity (1,000s of atoms in a protein) at the nanometer scale, in order to elucidate molecular structure and its relationship to biological function. Even more specifically, within the context of the scientific story being told, what is the main purpose of the planned figure? If a figure attempts to serve too many purposes, it may become cluttered and ineffective. In articulating the purpose of the figure, bear in mind the target audience (Box 2) and the minimum level of detail required to convey the scientific message. These points will help drive choices as to what MolVis approaches and software packages are best suited to the problem at hand.

**Box 2. Nine Simple Rules for Biomolecular Graphics**

Inspired by the *Ten Simple Rules* series [36], the following advice most closely complements previous collections devoted to publishing papers [37], making oral presentations [38], and creating good posters [39].

**Rule 1: Study the masters; be multidisciplinary.** Study the molecular artistry of legends such as Geis [40], and also note that many useful areas are only tangentially related to traditional biosciences. These include the theories of statistical graphical design and data/information visualization, as considerably advanced by the likes of E. Tufte [41] and the late J. Tukey. Therefore, be adventurous and sample these other areas too.

**Rule 2: Emulate the masters; be opportunistic.** Geis, Goodsell, and others provide tangible examples of what to strive for. A useful starting point is to emulate the principles illustrated by their masterpieces, selectively incorporating elements of their designs in approaching your own images. Similarly, if you spot useful methods in unrelated fields (e.g., a rep style from statistics), adapt that for good use in creating your own graphics.

**Rule 3: Be clear and consistent.** Clarity is a virtue. Understanding an illustration should not require detective work by the reader. This requires care in creating both the images and the accompanying legends. A corollary of this rule is to be consistent in creating figures (Box S1 in Text S1, Tip 2). A manuscript with several 3D images will be more user-friendly if a canonical orientation is defined early on, and subsequent views are defined with respect to that. Similarly, introduce clear symbolic and diagrammatic conventions early in the text, and adhere to them throughout.

**Rule 4: Prioritize figures, plan ahead.** Cleverly crafted images are a powerful form of information compression—a single figure can convey more meaning than pages of text. Thus, place just as high a premium on the quality and clarity of illustrations as on the scientific text itself. A possible rule of thumb is that at least as much time should be spent per figure as is spent writing two pages of text (assuming ∼500 words/pg). First-rate molecular graphics are the cornerstone of many high-quality publications, and require considerable patience and planning.

**Rule 5: Careful with captions.** Captions should not be overlooked. A well-written caption that accompanies a useless graphical panel will likely come across as an afterthought. Conversely, a beautiful, information-rich image lacking a correspondingly high-quality caption is hardly more informative than a random array of pixels. First-rate biomolecular graphics require that sufficient effort be dedicated to this often-overlooked part of the figure.

**Rule 6: Have others critique your illustrations.** Have others peruse your figures (while still works in progress), with an eye toward what can be improved, what is unclear or missing, etc. This will enhance the pedagogical value of your illustration, making it lucid to more readers. Doing so earlier rather than later will avoid potentially wasting time on what is shaping up to be an obtuse or unclear figure. Similarly, peruse the literature and note particularly bad or unclear artwork, poorly designed illustrations, opaque captions, etc.; most importantly, study these figures to pinpoint what you find to be their shortcomings, and avoid those pitfalls in your illustrations.

**Rule 7: Tailor to the task or audience at hand.** The graphics in a definitive, 20-page tome may not be optimal for a concise, four-page report aimed at a general audience. Similarly, figures will likely need to be reformulated for effective use in poster or oral presentations, versus manuscripts. Strive to match illustrations with the intended audience/purpose; the burden of doing so lessens over time, as you gradually accrue a library of raw images and figure panels for use in creating new slides, posters, etc. Bear in mind certain best practices that maximize the audience to which your graphical artwork is accessible (e.g., employ color charts and texture maps to make images that are interpretable by colorblind viewers).

**Rule 8: Embrace state of the art tools.** Rather than stick with familiar or convenient tools (e.g., what a labmate showed you how to use a few years ago), experiment with new methods and software. The initial effort invested in learning a feature-rich package (PyMOL, VMD, etc.) will be repaid manyfold once you've scaled the learning curve. This Rule applies to both software *and* hardware—embrace the latest technologies, such as GPU-accelerated graphical rendering, learn about sophisticated methods like ambient occlusion lighting [42], and so on.

**Rule 9: Learn to script.** To be poised to act on the “dig into the code” philosophy of Rule 8, note that you will vastly expand your graphical horizons by learning programming or scripting languages compatible with the API of your favorite graphics packages (if PyMOL then Python, if VMD then Python or Tcl, etc.). As a first step to learning languages such as Python [43], begin writing scripts in the command language of the vis software (PyMOL macro files). Box S1 in Text S1 offers practical advice on doing this. The many advantages of scripts include automation, and the fact that they make the figure-creation process “self-documenting” (so images can be exactly reproduced, tweaked, etc. at a later date).

The next step—selecting the optimal tools from among the myriad available software packages—is often quite difficult. Multiple tools are often necessary in the workflow leading to a finalized piece of molecular graphics. For instance, electrostatic potentials may be computed in a pre-processing stage, followed by actual scene construction and rendering of grid maps in PyMOL or VMD, and then post-processing in a graphics editing application such as Adobe Photoshop (Box S1 in Text S1). A comprehensive review and comparison of MolVis software suites is beyond the scope of this primer; such useful information has been tabulated recently by Bottomley and Helmerhorst in 2009 (see Table 9.2 in [2]), and by O'Donoghue et al. in 2010 (see Table 1 in [6]). Also, the Protein Data Bank (PDB) offers a thorough, annotated compilation of software [7]. Some of the existing software packages are more monolithic than others, but none is so fully featured that every conceivable MolVis task can be achieved using it alone. Features of an ideal software suite include (i) an active user community (useful in times of trouble); (ii) cross-platform interoperability (Linux, Mac, Windows; an important factor influencing the development of Java-based programs such as Jmol [8] and ProteinShader [9]); (iii) standards compliance and open-source licensing; (iv) a built-in, documented application programming interface (API) and scripting capabilities; (v) extensible and flexible; (vi) robust; and (vii) feature-rich (e.g., good built-in font support for labeling atoms). No MolVis package meets all of these exacting criteria, but PyMOL and VMD [10] have achieved broad popularity (substantial user bases) because of their performance against many of these measures; indeed, these two programs were used to create all the figures and animations that are cited in this tutorial.

## Two Particular Tools: PyMOL, VMD

MolVis programs can be classified into two fundamentally different types: stand-alone software and Web-based tools (applets, plug-ins, or remote/server-side programs). This distinction may eventually be blurred by the increasing availability of 3D structures, the growing need to integrate structure/function data, and the rising pervasiveness of online publishing; for instance, the electronic journal *PLoS ONE* now offers integrated “iSee” datapacks that augment published articles with interactive, 3D structural viewing capabilities [11]. The present guide focuses on stand-alone software, but most of the ideas (Boxes 1 and 3) and practical advice (Box 2, Text S1) are also applicable to figure creation via web-based tools. A recent discussion and catalog of online tools and servers can be found in reference [2].

**Box 3. Typical Graphical Tasks**

**Ligand-binding sites.** These detailed (Å-scale) illustrations focus on local 3D structures of active sites, ligand-binding pockets, inter-atomic interactions, etc.; text labels are often used to identify particular atoms, bonds, distances, motifs, or other relevant structural features.

**Overall fold.** The fold of a nucleic acid or protein domain is most often displayed in the popular ribbon or cartoon representation. Combined with depth-cueing, the highly schematic ribbon style is ideal for showing the layout of 2° structural elements in 3D space.

**Structure comparison.** A common approach to compare 3D structures is to align domains by superimposing coordinates, either pairwise or as a multiple structural alignment. Side chains are generally omitted from such reps to reduce clutter, and the backbone is drawn either as a C_α_ trace or ribbon cartoon; for complex superimpositions to be interpretable, the proteins should not be too dissimilar (<3 Å RMSD) or overly complicated. Structure comparison is used both when sequences and structures vary (superimposing a family of homologs), as well as in simpler problems involving only conformational differences (NMR bundles, MD trajectories).

**Interfaces.** Biological activity (catalysis, signal transduction, *etc.*) results from detailed interactions at molecular interfaces, possibly involving proteins, nucleic acids, or small molecules. Suitable representation styles for interfaces are wireframe or ball-and-stick (if zoomed-in on part of the interface), or a space/volume-filling method (ASA, spheres; if zoomed-out such that the entire interface is visible). It is crucial to distinguish (via coloring, labeling) individual surfaces participating in the interface, as well as noncovalent interactions between atoms mediating the interface. This type of figure also includes related features of protein topography, such as cavities, channels, surface ridges, and clefts (Section 2.5 of Text S1).

**Higher-order structures.** Oligomers and higher-order polymers are inherently difficult to illustrate because they occupy a length scale intermediate between the atomic/molecular (where traces, ribbons, etc. are used) and the µm-scale structures of cell biology (where schematics or simple diagrams suffice). Large complexes can be simplified by rendering subunits as surface envelopes, using a large probe radius to create a low-resolution image of the assembly. If the intent is purely to show the architectural layout of subunits, consider the highly schematic approach of drawing subunits as simplified objects (polygons, ellipsoids, etc.) that represent the overall shape of each subunit.

**Volumetric datasets.** This type of data varies continuously over 3D space, and therefore poses several illustration challenges. Electron density is one of the most familiar types of volumetric data; other common forms include “derived” physicochemical properties such as electrostatic potentials (see Text S1, Video S2, and Figure S4).

**Conceptual covers.** An aesthetically pleasing cover is perhaps the most difficult type of graphic to create, and will likely involve an extensive amount of inventive post-processing of raw graphics. In contrast to figures in journal articles, the fundamentally different purpose of a cover graphic enables—and maybe even necessitates—a greater degree of artistic license [44] to be exercised.

Among stand-alone programs, PyMOL and VMD are well-established in structural and computational biology, and can be used to launch other programs. For instance, a user-written interface to the APBS [12] electrostatics solver now exists as a “plug-in” for PyMOL [13]; similarly, VMD can be used as a graphical front-end and visual analysis tool in conjunction with molecular dynamics (MD) codes such as NAMD [14]. Most important for those planning to advance from novice to expert user, both software suites offer a feature-rich API, in either the Python (PyMOL and VMD) or Tcl (VMD) programming languages. Several aspects of the PyMOL API are illustrated in the scripts used to create the following series of case studies (scripts are available online as part of the accompanying PyMOL wiki site).

### Case 1: Domain level; overall fold [≥novice]

**Figure 1:**
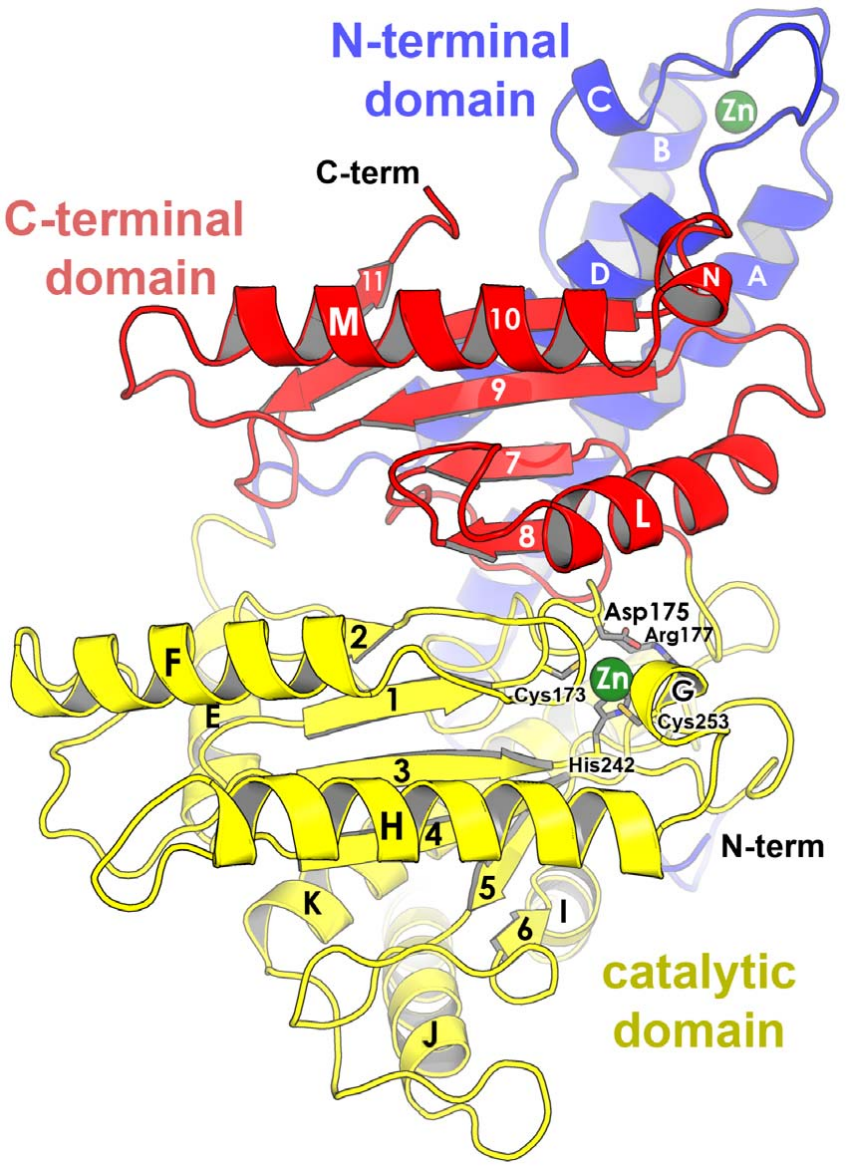
The overall fold of a carbonic anhydrase. The three domains of CsoS3 (PDB 2G13; [21]) are distinguished by separate coloring (blue, yellow, red). The orientation was chosen to feature the location of the active site (outlined by side chains and zinc ion), and to show the two-fold symmetry relationship between the active domain (yellow) and homologous but defunct domain (red). Structural elements are labeled directly on the individual SSEs. Domain labels are colored to correspond to the domains they are labeling. Labels in the active site are given an “outer glow” to make them legible in a region of the figure that is dense in detail. Depth is conveyed by use of fog, veiling less important structural features toward the back of the enzyme.

The overall fold is often the first figure in a structural report, usually depicted as a cartoon ribbon. Figure 1 shows an example of this type of graphic. This figure aims to depict and clearly label the domains and 2° structural elements (SSEs) comprising the protein. As the key to understanding the relationship between structural elements and protein function, it will be a figure of repeated reference for the reader. There are four critical goals in constructing such a figure: to optimally orient the molecule, choose between stereo or mono representation, settle on a meaningful coloring scheme, and clearly label relevant structural entities.

The molecule should be oriented so as to minimize unnecessary overlap of SSEs. Neglecting to do so will make it difficult to perceive the relative depth of the overlapping features and the overall fold. Ideally, the viewer should be able to visually trace the path of a protein chain from N′ to C′ terminus without ambiguity. Finding this “canonical” orientation can be difficult, especially for large proteins or complexes, so survey many options. As if these are not enough constraints, one must also consider the layout of later figures. Subsequent figures may involve close-up views of the active site, protein interaction interfaces, or other areas of special interest. Therefore, if at all possible, orient the molecule in this initial figure so that spatial relationships among these important sites are clearly illustrated too. If chosen carefully, it will be possible to minimize the total number of different orientations of the molecule illustrated in the paper, thereby minimizing the chance of spatial disorientation and confusion of the viewer. In many cases, no more than two orthogonal orientations of the molecule will be required for the whole paper. The relationship between the two orientations should be clearly labeled by a curved arrow that shows the direction and magnitude of rotation (Figure S2G), or else the relationship should be explicitly stated in the legend. If terms such as “front view,” “top view,” “side view” are included in the text, then also include these labels in the figure so that the reader can become properly oriented with just a quick glance.

Split stereo views greatly aid depth perception (Section 2.7 of Text S1). It is simple to make a stereo figure in PyMOL: chose an orientation, activate stereo (using the “stereo” command), and render the image. Unfortunately, not everyone is able to perceive stereo, and it is sometimes inconvenient to find stereo glasses. Instead, a pair of orthogonal views can convey the perception of depth without the need for glasses or the training in how to view stereo. Whether or not stereo is used, shadowing and fog supply valuable depth-cues. (These features are activated by default in PyMOL.) The effect of fog is enhanced by adjusting the front and rear clipping planes (Box 1) so they touch the front and rear of the molecule, respectively. Features in the rear of the molecule will be lightly veiled in fog, while features in the front will be sharp, crisp, and bright.

As a general rule, figures showing the overall fold (Figures 1, S2, and S3A) should label each SSE, domain, or any other structural element of special interest (such as features referred to in later figures or text). Labels should be large enough to be clearly visible, but not so large that the figure looks cluttered. Labeling can be done in PyMOL, or via graphical editing software (e.g., Photoshop or Illustrator) for more fine-grained control of label placement, font, color, and size. If possible, label helices and strands directly on the elements themselves, as in Figure 1. In some cases it might be necessary to place the label a short distance away from the SSE, but this should generally be avoided since it can become ambiguous to the reader to which element a label refers; it may be necessary to increase the thickness of the helix or strand to accommodate direct labeling. For large (e.g., multi-domain) structures, consider simplifying the figure by portraying helices as cylinders instead of ribbons (the “cartoon_cylindrical_helices” setting in PyMOL). If the shading or lines of the SSE obscure the legibility of the label, it is possible to impart an “outer glow”; this creates a small halo of white color around the label, thereby better contrasting it against the background. For stereo figures, take special care to place the labels so they appear at the same depth as the feature being labeled. While PyMOL renders labels in stereo at proper depths, one does this manually in Photoshop: First, place labels in one of the panels, either left or right. Copy the labels to the other panel so they are at the exact same height as the corresponding labels in the original panel. Put on stereo glasses. Then, simply adjust the horizontal position of the labels in the left panel until they appear to be at the same depth as the feature being labeled.

**Figure 2:**
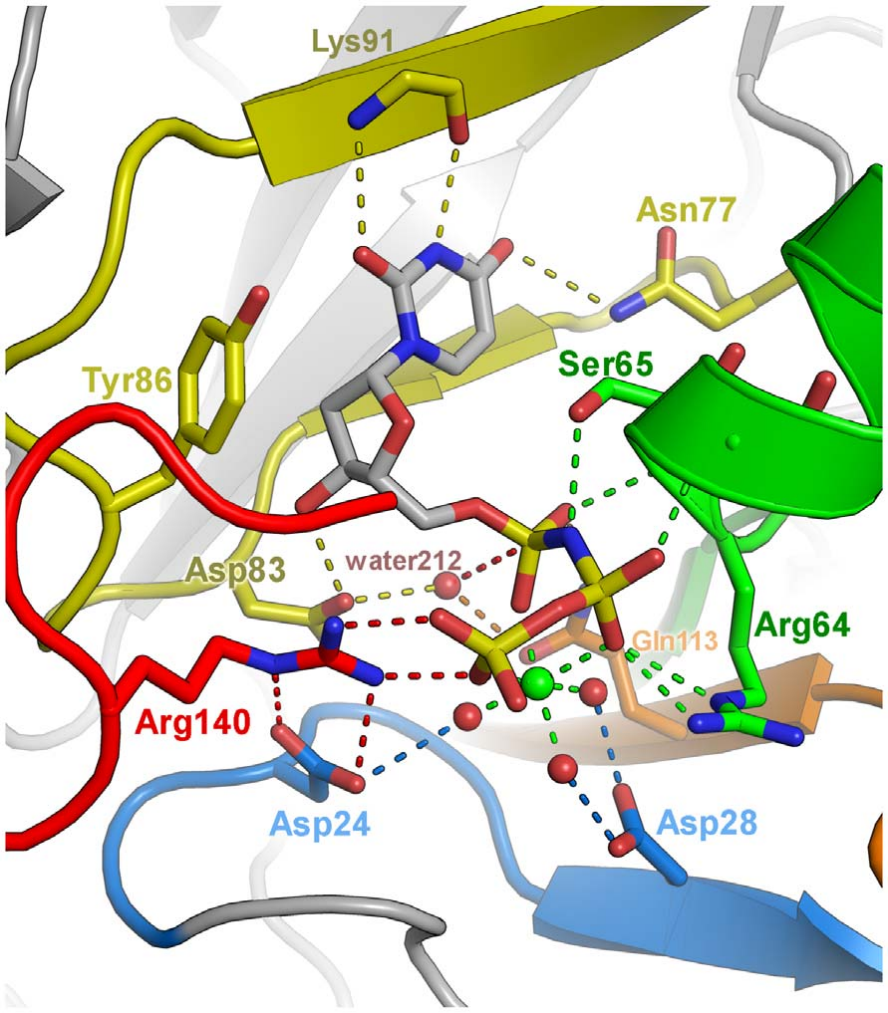
The active site of *Mycobacterium tuberculosis* dUTPase. The orientation of the active site of this dUTPase (PDB 1SIX; [35]) was chosen to feature the geometry of the reaction it catalyzes, specifically the in-line nucleophilic attack of water 212 on the alpha-phosphate of dUTP. The orientation was fine-tuned to eliminate overlap of side chains and to make all hydrogen bonds (dashed lines) visible. Only side chains directly involved in catalysis are depicted. Carbons are colored according to five conserved motifs of the dUTPase family. Non-conserved residues are given a less-distracting gray color and are veiled in fog. Label colors match the side chains being labeled.

Strive to limit the number of colors used in the figure of the overall fold. A simple grayscale can be sufficient to convey depth, avoid journal color charges, and reproduce accurately on black/white photocopiers (see also Box S1 in Text S1). For multi-domain proteins, a separate color is often assigned to each domain (Figures 1 and S3A). If a color scheme is chosen for this figure, adhere to this scheme in later figures so that the reader can easily recognize how structural elements relate to one another, in space and in position along the amino acid sequence. If the structure described is related to a previously described structure in the literature, consider adopting the existing color scheme; this will minimize confusion, and readers familiar with that literature will appreciate the continuity of convention. If the illustrated structure is a new fold, the chain is often color-ramped so that successive structural elements (domains or SSEs) are graded from blue (N′)→red (C′). Such a scheme is helpful in distinguishing, at a glance, whether a particular structural feature arises near the beginning, middle, or end of the polypeptide chain. In depicting multi-subunit assemblies, consider separately coloring each subunit, employing particular coloring schemes for homo-/heteromeric complexes, etc.; doing so is especially helpful for intricate quaternary structures, such as domain-swapped oligomers [15]. An example of a ribbon cartoon for a moderately complex dimer, alongside its 2D topology diagram, can be found in Figures 2 and 3 of ref. [16].

### Case 2: Ligand-binding sites [≥novice / intermediate]

Evolution has molded ligand-binding sites to perform specific and unique functions, giving a protein its identity, and often its name. It follows that figures portraying ligand-binding or active sites (Box 3) are often the primary focus of the results and discussion sections, and therefore should be as clear as possible. However, lucidly illustrating an active site can be more difficult than showing the overall fold, because optimal renditions of binding pockets often include both low-resolution (cartoon) and high-resolution (wireframe/stick) representations (“reps”), as exemplified in Figure 2. In addition, many text labels are often required to describe a ligand-binding site in terms of particular atoms, residues, SSEs, dashed lines, and associated distances (hydrogen bonds, ionic interactions, etc.). Beyond the examples provided in this guide (Figures 2 and S3B and S3C), literature examples include (i) a combination of wireframe, stick, sphere, cartoon, and surface reps for the ligand-binding site of a hexamer (Figure 7 in [17]); and (ii) ion-binding sites in DNA portrayed via a mixture of cartoon tubes, spheres, sticks, and isocontour surfaces (Figure 4 in [18]).

As a first step in binding-site figures, take care to eliminate structural features that are irrelevant to ligand binding. Begin constructing this figure by displaying only the ligand and the residues directly involved in binding (e.g., restrict atom selections to a ≈5–10 Å neighborhood about the ligand). Typically, these atoms are rendered as sticks or ball-and-sticks (Figure 2). Residues that connect the ligand binding residues can be shown as cartoon, or not at all. To reduce clutter, the four main-chain atoms (N, C_α_, C, O) should not be drawn as sticks, unless they interact with the ligand too. It requires time and patience to specify which residues are shown and which are not, and this choice may be optimized more efficiently using a script rather than interactively—a script is easily edited and re-run, and makes a figure easy to reproduce in the distant future (Box 2, Rule 9). Binary files such as PyMOL sessions (“.pse” suffix) also can be saved for reuse, but are not as amenable to future modification as are (plaintext) scripts.

As with the overall fold, it is important to find an orientation of the ligand-binding site and its proximal environment that avoids overlapping side chains, ligand atoms, or other relevant structural features. It is also helpful if the orientation of the active site is similar, or at least explicitly relatable, to the canonical orientation used to portray the overall fold (see, e.g., the green arrow “attached” to a β-hairpin to help orient the viewer in Figure 7 of [19]). By default, standard colors (oxygen, red; nitrogen, blue; etc.) should be used in these atomistic illustrations; note that carbon is often colored white, gray, or green. Because of the general crowdedness of binding sites, judicious use of depth-fog or stereoviews can greatly help in depicting these structures. If shadows cast distracting patterns on important active site side chains, then consider disabling or modifying the shadowing effect.

### Case 3: Structural comparison [≥novice / intermediate]

**Figure 3:**
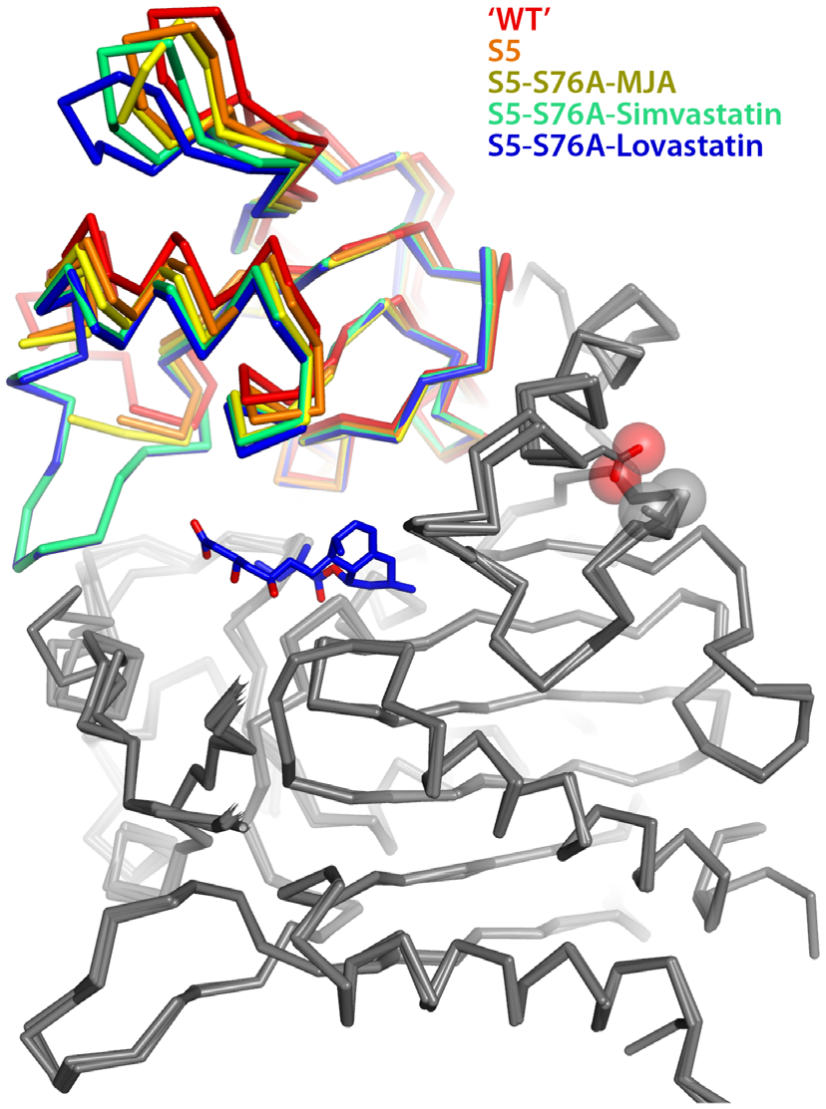
An overlay of five simvastatin synthetase crystal structures, illustrating varying degrees of hinge closure imparted by ligand binding. Hinge motion in this two-domain enzyme (PDB 3HLB, 3HLC, 3HLE, 3HLF, and 3HLG) is highlighted by superimposing only atoms in one of the domains (colored grey in this figure). The range of motion is highlighted by the rainbow colors assigned to the upper domain. The orientation of the molecule is chosen to make the range of motion evident (hinge axis normal to the plane of the page). Each of the structures is labeled explicitly in the figure, rather than burying the information in the figure legend. Color coding the labels makes it easier to comprehend how each ligand affects the hinge motion. The structures are represented as C_α_ traces rather than cartoon ribbons because the motion is relatively small, and the C_α_ trace allows a more exact representation of the position of the atoms.

Structures are often compared by superimposing C_α_ atoms of two or more molecules, as illustrated in Figure 3. Properly constructed, these versatile figures can be used to convey bioinformatic information for a protein family (evolutionary distances) or structural and dynamical results for a single protein, such as conformational heterogeneity in an NMR bundle, structural changes inferred from different crystal forms, or thermal motion computed from MD simulations. The procedure occurs in two stages: establish equivalencies between positions in the two structures (A, B), and then compute the alignment so as to optimize some function (e.g., minimize sum of least-squares differences in positions of paired atoms). Note that both the calculation and graphical illustration tasks are greatly simplified if A and B are merely different conformations of the same molecule (or simple point mutants, as in Figure 3). Also, pairwise comparisons are more easily illustrated than multiple structure alignments. For multiple structures superimposed to show a progression (e.g., hinge motion), it may be helpful to assign colors in a stepwise gradient; for instance, the most closed hinge is black, the most open hinge is white, and intermediate states are gray. This scheme can also work with rainbow colors (Figure 5 in [20]). In large structures, individual domains should be colored separately. Only when the structures are highly divergent should one consider using cylinders to represent helices, as cylinder orientations are highly dependent on exact residues included in the helix definition. (Though often assigned via pre-processing in a 2° structure calculation program, note that residue structure classifications can be manually altered in PyMOL.)

The clearest way to represent superimpositions depends on the degree of 3D similarity and the size of the structural unit. Single domains with small coordinate RMSD (<2 Å) should be superimposed as C_α_ backbone traces rather than cartoons, because a trace will be interpretable at this high level of similarity and is more precise than cartoon renderings. Cartoons are more helpful than backbone traces when superimposing entities (single- or multi-domain) with large RMSDs, exceeding ≈2–3 Å. The smooth cartoon eliminates some of the structural details that would otherwise obscure or distract from the relevant, larger-scale differences. In this respect, it is preferable for multi-structure alignments to use thin, spaghetti-like cartoons—these are well-suited to showing 3D alignments of homologous protein structures (Figure 1B in [21]), frames periodically sampled from MD simulations (Figure 3 in [18]), and regularly spaced (interpolated) structures generated by projection along the principal components of a dynamics trajectory (Figure 2 in [22]). Note also the strategic use of color in some of these illustrations—structural regions that are irrelevant or virtually identical can be de-emphasized by coloring them uniformly in a subdued hue (e.g., light grey domain on a white background).

### Case 4: Volumetric data, surface mapping [≥intermediate]

Volumetric properties vary as a function of 3D position (***r***), in and around a molecule. Depending on the physical property, volumetric data may be scalar-valued (e.g., electron density, *ρ*(***r***)) or take the form of a vector field (e.g., electrostatic field). This is one of the basic difficulties of rendering volumetric data: It is continuous through space, unlike discrete molecular entities such as atoms or bonds, and is therefore less amenable to representation via geometric primitives. The other intrinsic difficulty lies in representing higher-rank tensors in 2D formats, such as a computer display or illustration. To address this well-known [4], [23] problem, volumetric data are usually represented as isovalue meshes or surfaces/contours. For instance, isocontour “chicken-wire” maps are indispensible for building a structure into electron density, and illustrating the agreement between final 3D model and crystallographic data. (Though not strictly volumetric, many “derived” physicochemical or bioinformatic properties [surface curvature, polarity, residue conservation, diffusional accessibility, etc.] can be mapped onto similar surfaces or meshes.)

Electron density is typically illustrated as a mesh surface (Figure S4A). A mesh is used rather than a solid surface so that one can evaluate the fit of the density to the underlying atoms. Appropriate contour levels are ≈1.0σ for 2*F*_o_–*F*_c_ maps and ≈±3.0σ for *F*_o_–*F*_c_ maps; those values should be specified in the legend. Positive contours are conventionally shown in shades of green, blue, or purple, and negative contour levels are generally colored red. When showing both positive (*P*) and negative (*N*) contours, avoid misleading the viewer by choosing values such that *P* = −*N*.

**Figure 4:**
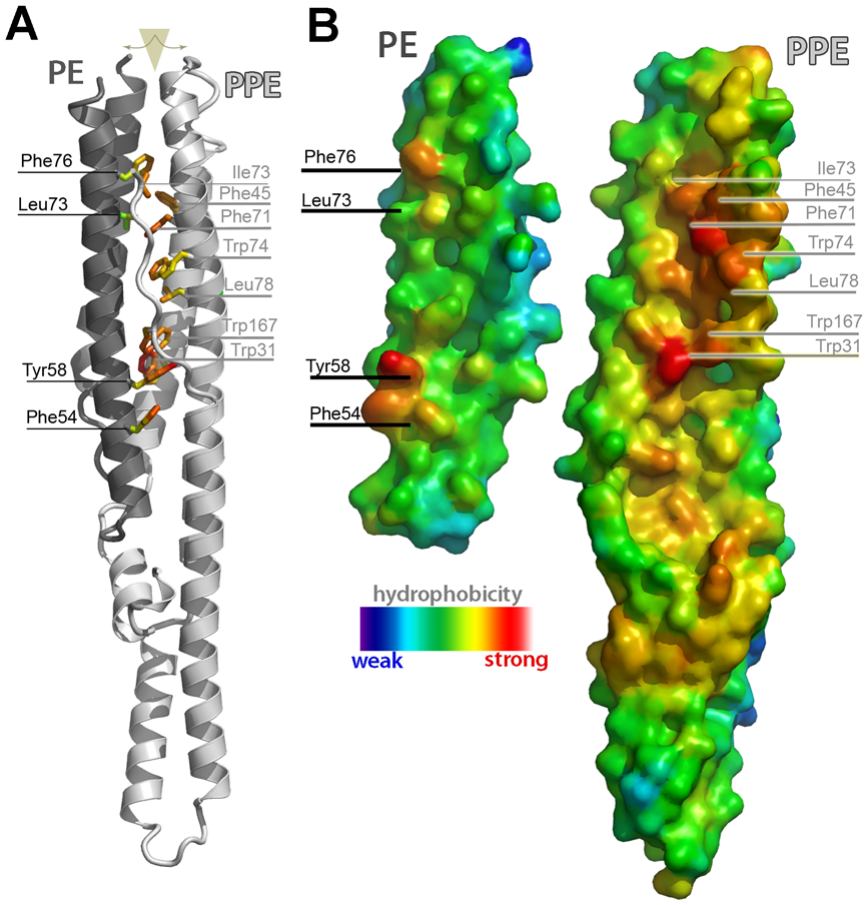
An intermolecular interface from the *M. tuberculosis* PE-PPE system (PDB 2G38). The complex between PE and PPE is shown as a ribbon cartoon (A); the two proteins are colored separately to make the interface evident. Hydrophobic side chains involved in the interface are labeled. The complementary surfaces are illustrated more clearly in (B), by splitting the complex apart like a clamshell (triangular wedge in A). Labels identify the PE and PPE proteins. Hydrophobicity is indicated by grading of cool→warm colors, as shown. Both panels are labeled and depicted on the same scale, so readers can easily see how 3D residue positions at the interface (A) correspond to apolar surface patches in (B).

Biophysical properties are most often rendered as a gradient of colors mapped onto a molecular surface (Figures 4 and S4). The value of a given property at each point on the surface is encoded by a color that can be interpreted by a key given in the figure. A surface is used rather than a mesh, because it is continuous and so better suited to illustrate the gradient of colors, uninterrupted by the holes of the mesh. The use of a surface does, however, conceal the atoms beneath, making it difficult to know what residue lies below a given point on the surface. It is tempting to make the surface semitransparent in order to see the hidden 2° structure cartoons or stick representations. However, overlaying many objects and surfaces can be difficult for the viewer to interpret. Thus, it may be best to forego transparency and label the positions of important residues at the surface using arrows, or show a 2° structure ribbon diagram side-by-side (same scale and orientation). As with electron density, positive (*P*) and negative (*N*) isocontours of physicochemical values generally should be scaled so that *P* = −*N* (e.g., −10↔+10 *k*_B_T/*e* for electrostatic potentials).

### Case 5: Interfaces [≥intermediate]

Like ligand-binding sites, molecular interfaces are challenging to illustrate because of their inherent structural complexity. Examples are shown in Figures 4 and S2F. Difficulty arises from the need to show two or more molecular surfaces as well as stick or wireframe reps of the atoms in contact across the interface. If the interface is small or mainly one-dimensional, then a single panel is sufficient (Fig. 4D in [24]). If the interface is large or two-dimensional, multiple panels may be necessary, each illustrating a different slice through the interface (Figure 5A in [24]). To help distinguish the two molecules, the surface of one molecule should be colored differently than the other. Another alternative is to split the interface apart like a clamshell, exposing both sides of the interface to the viewer; this technique is employed in Figure 4B. Although it does not reveal surface complementarity, another approach is to show one molecule with an overlaid surface and the other as a bare cartoon in front of the first (Figure 3D [25]).

### Case 6: Higher-order structures [≥intermediate/advanced]

**Figure 5:**
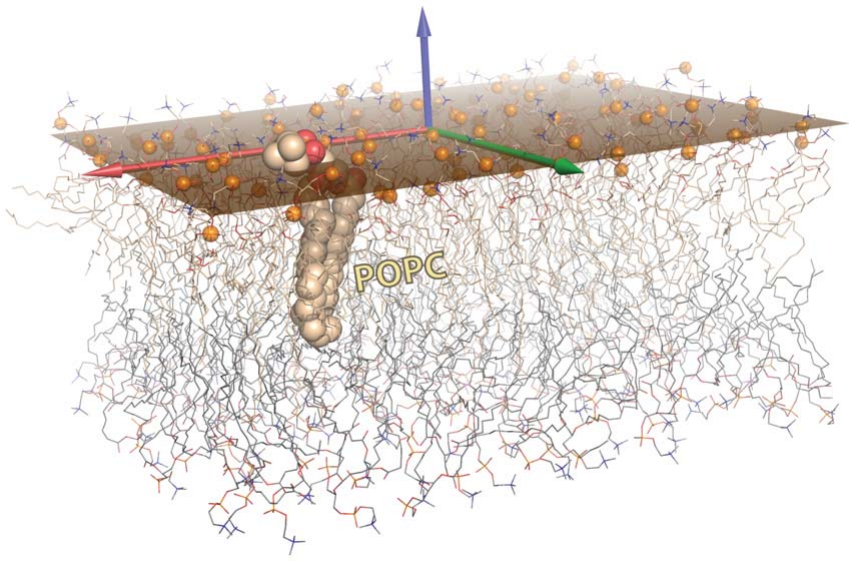
Best-fit planes. This figure illustrates the results from computing and rendering the best-fit plane to a set of atoms. As described in the text, the plane was calculated via SVD on the phosphate atoms (orange spheres) of one leaflet of this POPC bilayer, and is rendered as a semi-transparent orange surface. The two leaflets are shown as wireframes, with a single lipid shown as CPK spheres; carbons are colored wheat in one leaflet and light grey in the other. The computed bilayer normal is drawn as an arrow, as are the two basis vectors which span the subspace defining the planar membrane.

Higher-order structures consist of many subunits assembled into a large-scale complex, possibly spanning hundreds to thousands of ångströms. Common applications include illustrations of crystal packing, large complexes (ribosomes, viruses) or multi-domain proteins (antibodies, Dscam), and biological assemblies such as cytoskeletal filaments and lipid bilayers (Figure 5). A common purpose of these images is to show the relationships between domains or molecular subunits, not atomic interactions (Figure S1). For these reasons, the molecules are typically rendered more schematically, with far less detail shown. Only for polymers of very small molecules should stick representations be used (Figure S3A in [26]; Figure S3 in [27]). Cartoon representations can be used, but care should be taken to eliminate complex shadowing; not doing so may render the ray-tracing step computationally infeasible, and may degrade the appearance of the final image by producing visual artifacts (particularly in periodic structures such as 1D polymers). Simplification should be introduced whenever possible. For example, smoothed loops and cylindrical helices may help simplify large proteins. At length scales exceeding ≈100–200 Å, entire molecules can be shown as molecular envelopes (e.g., the middle layer in Figure 3 of [28]). These envelopes can be calculated from atomic coordinates as the ASA, using an inflated probe radius so that the molecule is effectively viewed, for instance, at 20 Å resolution. Even more aggressive methods may be necessary in the ≈500–1,000 Å range, including, for instance, schematizing entire oligomers as polygonal plates and using color gradients as additional depth cues (Figure 3, [28]). Note that lighting and outlining effects can be tuned to further clarify the highly schematic renderings often necessary for higher-order structures. The sheer visual and computational complexity of higher-order structures drives the development of efficient multi-scale/multi-resolution approaches as a major current area of MolVis research. Though it lies beyond the scope and space limitations of the present tutorial, we note that this area is rapidly advancing in terms of basic algorithms and methodologies (e.g., [29], [30]) as well as practical tools (e.g., the Situs software package for multi-resolution structural work [31]).

### Case 7: Animations [≥intermediate]

If a picture is worth a thousand words, then a movie is worth a million. Though animations are more costly in terms of user effort and computing time (hundreds to thousands of individual images must be rendered as movie frames), nothing makes complex mechanisms clearer—see, for example, Video S1 or the helicase animations in ref. [32]. Movies fundamentally differ depending on whether the molecule is static or dynamic (Table S1)—one class shows only rigid-body motion (static coordinates, rotating camera), while the other types animate changes in atomic coordinates over frames (i.e., conformational motion). Tools like iPyMOL simplify movie-making by automating the process of interpolation between conformational states, and recent PyMOL versions provide enhanced functionality for constructing movie scenes, keyframes, and transitions. In some cases, improved 3D depth perception can be achieved by rocking back and forth 15°, while for other scenes a 360° rotation about the vertical axis may suffice. If the exact aim of the animation is quite intricate, complex modes may become necessary (rocking about multiple axes, successively or simultaneously). In general, 40 frames per revolution is sufficient; for conformational changes, 20 frames might be enough. It is helpful to have the movie end with the same frame as it starts, so that there is a smooth transition when the movie cycles to the beginning. Conformational dynamics (e.g., MD trajectories) can be combined with camera motion in the most complex type of movies, but simply making additional movies from alternative perspectives might be preferred in order to avoid viewer motion sickness. A variation on this theme is to construct the movie using scenes containing duplicate molecules that differ only in orientation (e.g., perpendicular views). The molecular motion is synchronized between the copies so that the trajectories progress identically; this is an effective means by which to visualize simultaneous events in the molecular dynamics of distant regions of a molecule (see, e.g., animations accompanying ref. [18]).

### Case 8: Compute and display a best-fit plane [≥advanced]

Advanced visualization projects straddle the line between molecular modeling and molecular graphics. For example, consider the task of computing and illustrating the best-fit plane to a set of atoms. Such a problem arises in many contexts, including membrane proteins and the lipid bilayers in which they are embedded. We address this problem (Figure 5) by starting with the PDB file for a bilayer composed of 200 POPC lipids (100/leaflet); our starting structure is the bilayer slab after equilibration via MD simulations [33]. The mathematical approach of singular value decomposition (SVD) was then used to transform the atomic coordinates into a new reference frame, defined by the three basis vectors that capture the underlying geometry of the bilayer. Most importantly, these three singular vectors correspond to the bilayer normal (*z*-direction) and the two vectors which span the 2D plane of best fit (“best” in the sense of linear least-squares minimization of the deviation of *z*-coordinates of all atoms from the plane). To represent this plane, we chose to display the minimal-area rectangle that contains all the planar projections of the selected atoms (Figure 5). Computationally, this method was implemented as two stages: (i) a lower-level Python module (“svdPLoS.py”) to perform SVD on an arbitrary selection of atoms (and related manipulations, such as coordinate transformations, computing planes as linear combinations of basis vectors, drawing PyMOL compiled graphics objects, etc.); and (ii) a higher-level PyMOL macro (“svdPLoS_fig.pml”) that “wraps” the Python code to create the actual image file—from PDB input, to SVD, to final ray-tracing. The scripts used to produce the final result (Figure 5) are available from the PyMOL wiki site.

## Conclusion and Outlook

Occupying a unique niche at the junction of computational, structural, and cellular biology on the one hand, and developments in computer hardware and algorithms on the other hand, biomolecular graphics has benefited greatly from the confluence of these two streams. Given the ongoing deluge of structural and bioinformatic data, this will likely continue to be the case in the foreseeable future. Despite recent advances, many visualization challenges remain, including (i) effective multi-scale approaches for ultra-large structures, particularly asymmetric ones such as the ribosome (see, for example, the approaches introduced in [29]), and (ii) vis methods that are simply *feasible* for tera- and peta-scale datasets arising with the recent advent of µs/ms-scale biomolecular simulations [34]. These two challenges are largely issues of computational efficiency. An even more basic problem is that of developing multi-“modality” representation methods: What new visualization and graphics tools can be invented to more effectively represent the intricate sequence ↔ structure ↔ function relationships uncovered by structural bioinformatics? For instance, a common approach to studying biomolecular electrostatics is to map potentials onto surfaces, followed by visual identification of highly charged regions of potential functional relevance (DNA-binding surface, cation channel, etc.). However, systematic comparison of physicochemical properties, such as potential maps, across a series of homologous proteins that do not exhibit perfect structural similarity is a far more difficult and ill-defined task than the analogous problem of structural comparison via superimposition. Advanced visualization and representation methods will likely play a role in overcoming such hurdles and enabling the next wave of breakthroughs in both biomolecular graphics and computational biology.

## Supporting Information

Text S1 Supporting information.
(0.27 MB PDF)

Figure S1 Biomolecular graphics in a nutshell.
(0.82 MB PDF)

Figure S2 Different representation styles and their relative utility.
(3.10 MB PDF)

Figure S3 A tetradecamer assembly: Overall architecture, bipartite domain organization, and Cd^2+^-binding sites.
(7.91 MB PDF)

Figure S4 Representations of volumetric data: Electron density and electrostatic potentials.
(3.29 MB PDF)

Video S1 Helicase animation. This animation shows the conformational changes in a helicase as it unwinds double-stranded DNA. The movie is of type M_d_V_s_ (using the nomenclature of Table S1), and was produced in animated GIF format using PyMOL and the scripts accompanying this primer.
(2.22 MB GIF)

Video S2 Electrostatics screencast. This screencast video is a step-by-step demonstration of the usage of PyMOL's APBS plugin to seamlessly integrate (i) the set-up and execution of a Poisson-Boltzmann electrostatics calculation with (ii) visualization of the resulting grid maps. The steps were performed on a GNU/Linux workstation, using relatively recent releases of the APBS (v1.1) and PyMOL (v1.2) packages.
(9.70 MB AVI)
