# Supplementary material for: An Introduction to Biomolecular Graphics
Source: PLoS Comput Biol. 2010 Aug 26;6(8):e1000918. doi: 10.1371/journal.pcbi.1000918 (PMC2928806; doi:10.1371/journal.pcbi.1000918)
Supplement: Text S1 — Supporting information. (0.27 MB PDF) [file pcbi.1000918.s001.pdf]

Supporting Information

The following materials are provided as supplementary information for *"An Introduction to Biomolecular Graphics"*.

- Supplementary Text (pg S2–9):
  - §1: Background & motivation for MolVis
  - §2: A review of MolVis concepts and principles
  - §3: Abbreviations used in this supplementary text
- Supp. Box S1: Practical tips for molecular illustrations (pg S10–11)
- Supp. Figure & Video Captions (pg S12–13)
- Supp. Tables (1; pg S14)
- Supp. Figures (4)
- Supp. Videos (2; online)
- PyMOL and Python scripts used to produce all the graphics and animations in this article are available at the URL `http://pymolwiki.org/PLoS/`.

Supplementary text

1) Background & motivation for MolVis

1.1) Biomolecular graphics in context

Biomolecular graphics is a subset of the broader field of molecular visualization and graphics, which is itself but a narrow slice of the general set of activities known as scientific / data visualization. The myriad scientific areas, subfields, and types of questions means that data visualization has traditionally employed a vast variety of modalities – including actual specimens, physical / tactile models, line graphs, 2D or 3D plots, bar or pie charts, photographs, drawings, movies, animations, and, most recently (since *ca*. 1970), computer-based visualization. [1] The first three-dimensional (3D) structural models of biopolymers in the 1950-60s marked the origin of biomolecular visualization as a distinct discipline – Watson & Crick's brass wireframe model of DNA and, subsequently, Perutz and Kendrew's wood or plasticine 'sausage' models of globins, stacked Plexiglas panes of electron density isocontours, and so on (see, *e.g.*, ref. [2], or Plate II and pgs. 239–266 of ref. [3]). While contemporary biomolecular graphics is largely computer-based, alternative modes of visualization (*e.g.*, tangible models or hybrid "augmented reality" approaches [4]) do exist and are highly effective. Also, note that the goals of molecular graphics activities occupy two related, yet distinct, categories: *(i)* visualization as a means of data analysis and exploration (*e.g.*, building atoms into electron density maps, visualizing structural transitions in a molecular dynamics (MD) simulation), and *(ii)* visualization with the end-goal being purely depictive (creating figures for publications, presentations, *etc.*). As the first area can be quite heterogeneous and largely project-specific, this primer focuses on the latter aim of generating graphical images that clearly and effectively communicate structure-related data.

1.2) Historical background & significance

Biomolecular graphics is motivated by the needs of biology, and is enabled by the tools of computer graphics. Advances in computer visualization and graphics technologies revolutionize molecular graphics every decade or so, while the fields of structural and molecular biology provide a steady source of increasingly sophisticated challenges that fuel the next cycle of development. For example, many molecular graphics developments during the 1970-80s were stimulated by the need to 'build' atomic coordinates into electron density maps in the course of structure *determination*; this is an ideal problem for interactive, 3D molecular visualization, and Jones' pioneering *FRODO* software [5] ushered in a new phase of computer graphics-based crystallographic structure determination (effectively displacing the mirror-and-Plexiglas-based Richards' Box [6] then in use). In terms of structure *analysis*, hand-held, physical models alluded to above (wireframe DNA, plastic proteins) were originally devised to visualize small organic compounds, perhaps ranging in complexity up to modestly-sized proteins such as insulin (though even this 51-amino acid polypeptide contains ≈ 400 non-hydrogen atoms). Such models are manageable for small molecules and biopolymers comprised of less than a few hundred atoms, but the challenges posed by the first macromolecular structures rapidly made it clear that *(i)* interactive visualization of coordinate sets and associated molecular properties (*e.g.*, electron density) would be vital in both determining and analyzing 3D structures, and *(ii)* existing approaches (rigid, immutable physical models) would be hopeless for even moderate-sized biomolecules. (It takes weeks to manually construct a model of the aforementioned insulin [1].) Therefore, the field of structural biology – and, now, structural bioinformatics – has provided both impetus and fertile ground for the development and application of novel computer graphics methods.

A pivotal step towards using computers to visualize biopolymer structures occurred in the 1960s, when Langridge and Levinthal demonstrated the power of computer graphics by interactively visualizing the early protein structures (globins, lysozyme) as wireframe models on an oscilloscope display [7]. Since those humble origins, molecular visualization (MolVis) and computer graphics (CG) have both rapidly progressed, with many synergistic interactions linking these two fields. For instance, the expensive, monochrome vector graphics hardware of the 1970-80s (*e.g.*, Evans & Sutherland display) was supplanted by colorized raster/bitmap graphics devices in the early 1990s (*e.g.*, SiliconGraphics systems)

and, eventually, by present-day, affordable workstations featuring CRT or liquid crystal displays. This shift from vector to raster/bitmap displays was followed almost instantly by the development of the raster-based *RasMol* molecular structure viewer in the early 1990s [8]. Having provided many technical advances in molecular 'scene' construction (*e.g.*, enhanced lighting, shading, and other depth-cues that became feasible because of the raster (*versus* vector) graphics display), *RasMol* became the first largely platform-independent and free MolVis software to achieve widespread distribution, and had the major impact of popularizing molecular graphics amongst biochemists. In a similar way, CG algorithms and MolVis software cross-pollinate and co-evolve: algorithmic developments in the CG community often translate into the next set of advances in MolVis and graphics software. For instance, ray tracing algorithms developed in the 1970s to create photorealistic images [9] were adopted by the MolVis community in the 1980s [10], and then achieved widespread use *via* popular codes such as *Raster3D* [11]. A most recent example of this phenomenon is the development of sophisticated ambient occlusion lighting schemes, and incorporation of these techniques into programs such as *QuteMol* [12] and ray-tracing libraries such as *Tachyon* [13]. These and many other hardware and software developments in CG have enabled scientists both to perform their research (*e.g.*, structure determination & analysis), and to communicate it in a variety of formats (illustrations for publications and poster presentations, animations for talks and online viewing, *etc.*).

Molecular graphics has benefited from a rich history of masterful illustrators, pioneers who thrived in the field's confluence of science & art. Examples from the pre-computer era include the late Irving Geis, who entered from a background in architecture and graphical design, and whose cogent artistry set him apart as an indisputable master in the first generation (1970–90s) of biomolecular illustrators [14]; indeed, Geis' legacy is such that the collection of his work has been purchased by the Howard Hughes Medical Institute as the Irving Geis Archives [15], and much of it adorns the pages of many textbooks. The subsequent generation includes such luminaries as David Goodsell, who draws from a background in computational and structural molecular biology; the 'Goodsell-style' molecular rendition – characterized by edge-enhanced outlining – is so effective that it has been adopted by some MolVis packages as built-in (VMD [16]) or user-configurable (PyMOL) display styles. More comprehensive reviews of the historical background and underlying principles of MolVis can be found in recent treatments by Bottomley & Helmerhorst [1], as well as accounts from the directors of two of the premier molecular graphics centers in the U.S. – Olson of the Scripps Molecular Graphics Laboratory [17], and Ferrin of the (Langridge-initiated) UCSF Computer Graphics Laboratory [18]. Finally, additional historical, conceptual, and practical information related to this discipline are available at several websites (*e.g.*, [19]), and various sources of information have been compiled at Martz & Francouer's online history of MolVis [20].

2) A review of MolVis concepts and principles

Several basic principles arise in practical molecular graphics activities (*e.g.*, '*anti-aliasing*'), and these concepts and terminology are also frequently encountered in the MolVis and computer graphics literature. Therefore, in addition to that which is presented below, note that many conceptual aspects of MolVis are treated in relatively recent reviews, including those of Bottomley & Helmerhorst [1], Goddard & Ferrin [18], and Olson [17].

2.1) Mode of operation

Molecular graphics software is operated in either active (*on-line*) or passive (*off-line*, *e.g.*, scripted) modes. On-line operation creates a scene *via* interactive manipulation of a molecule (mouse motion, menu clicks, *etc.*). Because real-time ray-tracing of complex scenes is not yet computationally feasible even for small proteins, off-line rendering is used to ray-trace a still image, or a sequence of scene images as frames for an animation.

2.2) Making a 'scene' – Geometry, graphical rendering

A '*scene*' *defines* everything necessary to produce an image: *(i)* what molecular entities are shown (which atoms, bonds, molecules, *etc.*); *(ii)* how each 3D object is rendered in terms of graphical '*primitives*' (Box 1), in a particular representation style (wireframe, ball-and-stick, CPK spheres, *etc.*); *(iii)* geometric

layout of the view (reference frames, clipping planes, camera position, perspective *versus* orthogonal projections); *(iv)* lighting, shadowing, and illumination; *(v)* transparency effects (*e.g.*, alpha-blending); and *(vi)* additional details, such as depth- (*z*-) buffering, texture mapping (see §2.6 below), text labels, and so on. One makes a scene by loading molecular objects into a session, and then manipulating settings, representation styles ('reps'), views, and so on.

The '*visualization pipeline*' is the series of computer graphics processing stages that transforms a molecular scene to the final 2D raster image displayed on the monitor. This process begins with a program's internal (software-specific) representation of a given scene in terms of geometric primitives and lighting models. That information is communicated to the necessary software stages and hardware processing units *via* the MolVis application's bindings to low-level graphical libraries (OpenGL, Direct3D), resulting in the necessary geometric transformations and final rendering / rasterization calculations by the graphics processing units (GPUs) found on most modern video cards [1].

The term '*rendering*' refers to the process by which the vis pipeline transforms a 3D molecular scene into the 2D (raster) image, a bitmap array of colorized picture elements called *pixels*. Rendering typically involves ray-tracing (Box 1), in which the properties of each pixel (hue, saturation, etc.) ultimately arise from the detailed interaction of each light ray with each of the potentially millions of geometric primitives comprising a scene. The sum total of each pixel's precise color assignment defines the image. Rendering is mathematically expressed by the 'rendering equation' of geometric optics [21], and ray-tracing is effectively a computational way for a MolVis program to approximate this equation for a specific, user-generated molecular scene. Further background on shading and related computer graphics (CG) concepts can be found in texts cited in [1], and in detailed reviews of advanced lighting and rendering methods [21].

2.3) Optimizing a scene – Lighting, radiosity, depth-cueing

Lighting, shadowing, reflectance, and depth-cueing are important factors to consider when optimizing a scene. Lighting is used to, literally, illuminate a scene. MolVis programs generally employ white light, and illumination can be adjusted in many ways, including *(i)* number of lights (two are activated by default in most MolVis programs, such as PyMOL and VMD), *(ii)* 3D locations of lights in a scene, and *(iii)* properties of the light sources. Light sources may be defined as either *positional* (point-source of isotropically-emitted rays) or *directional* (essentially a vector field of parallel light rays impinging on objects in the scene). Both materials and light sources can be further characterized in terms of their *ambient*, *diffuse*, and *specular* components (see below); these parameters are key elements of the radiosity/rendering equation, and therefore severely influence the final appearance of a rendered molecular scene. Shading and reflectance properties play key roles by dictating the geometric and physical interactions between incident light rays, emitted light rays, and the material properties of the geometric primitives from which scattering occurs, for each light ray that intersects an object in the scene. Thus, the final appearance of an illustration is a complex result of lighting properties, in conjunction with *(i)* actual scene geometry (geometric primitives [such as triangles] and their surface normals) and *(ii)* the '*material*' properties that dictate the interaction between primitives and impinging light rays (see below).

Depth-cueing refers to modification of the representational properties of objects as a function of their depth perpendicular to the screen. For instance, in a wireframe representation, a MolVis program in depth-cueing mode would render bonds that recede more distantly into the screen as narrower lines and, assuming a dark background, would also darken those bonds and atoms. This technique greatly enhances depth perception, and therefore three-dimensionality, of the scene. Additional approaches include *z*-clipping (depth clipping), usage of additional sources/types of lights, more sophisticated illumination and shadowing models (*e.g.*, ambient occlusion lighting [12]), color gradients, edge-cueing, perspective (*versus* orthographic) projection modes, motion and motion-blurring, and application of a diffusely-scattering atmospheric 'fog' that thickens towards the far ('*rear*') clipping plane.

2.4) Molecular representations

A basic difficulty of biomolecular graphics is that the relevant structural features often span many length scales, ranging from atomic (Å-scale details of antibiotic binding in a ribosomal active site) to organellar

(*e.g.*, protein/RNA organization in ribosome subunits) and even cellular-scale ultrastructures (ribosomes tethered into polysomes; cytoskeletal tubes, filaments, sheets). Conventional molecular representation styles ('*reps*') can be classified by level of detail (Fig. S1), and standard reps exist for both molecular structure (wireframe lines, space-filling spheres, ribbons, *etc.*) and associated properties (electron density meshes, electrostatic potential isocontours, *etc.*), as outlined below and detailed in [22].

High-resolution data, such as active sites (Figs S3B, S4A) or detailed atomic contacts at an interface (Figs 4, S4), are often best-captured by '*wireframe*' representations, where covalent bonds are drawn as lines and atoms are implied at vertices. Inspired by early physical models of small molecules, variants of the wireframe style include '*stick*' models ('*licorice*' in VMD), as well as the slightly more elaborate '*ball-and-stick*' reps (Fig. S4C). Inflating the radii of the balls to reflect actual atomic (hard-sphere) volumes yields spherical '*space-filling*' reps. These renditions are often called '*CPK*' or '*vdW spheres*', and the union of covalently-bonded atoms is the molecular *vdW envelope* (Fig. S2E). Because essentially all of the electron density is encompassed within the vdW radii, these highly-detailed models accurately represent molecular shape, and are therefore useful in, *e.g.*, gauging steric complementarity between the potential binding site of a protein receptor for a small-molecule ligand, as is done in the field of docking and structure-based drug design. Spheres are generally colored by element type, using the *Corey-Pauling-Koltun* (*CPK*) convention that H = white, C = black/grey, N = blue, O = red, P = orange, and S = yellow.

The structural fold of a biomolecule is typically illustrated using backbone *ribbon*, *cartoon*, and *trace* reps (Fig. S2). In a *trace*, only one atom per residue is shown (backbone C_α of proteins, phosphorous of nucleic acids), and atomic positions are connected by line segments. The lack of atomic detail allows one to follow the connectivity of 2° structural elements (SSE). Ribbons and cartoons are similar to traces, except that straight lines are replaced by smoothly-varying splines (see below) that approximate the biopolymer backbone. Helices are represented as thin spirals (in backbone cartoons), thicker ribbons, or solid cylinders, while β-strands are drawn as thick arrows that indicate N′→C′ chain directionality. By eliminating atomistic clutter, these styles clearly show 2° structural elements (SSEs), as well as the connectivity and topological arrangements of SSEs relative to one another (*i.e.*, the *fold*). Introduced by Geis & Dickerson [23] and systematized by Richardson [24], ribbons have become the most widely popular style of 3D structure portrayal. Note that these terms are often used loosely – *e.g.*, the above definition of '*trace*' is termed a '*ribbon*' in PyMOL, and '*ribbon*', '*cartoon*', and even '*ribbon cartoons*', are frequently used interchangeably [1].

2.5) Molecular topography: Surfaces, voids, *etc.*

Physicochemical features are often represented *via* molecular *surfaces* or *envelopes* (Fig. S2F). These renditions are akin to space-filling spheres in that they impart a sense of overall molecular volume, shape, and surface properties (charge, acidity, curvature, shape complementarity [25]). There are many closely related constructions. The solvent-accessible surface area (SASA) is related to CPK spheres *via* the classic Lee & Richards "rolling ball" molecular surface [26] (and related algorithms, such as that of Shrake & Rupley [27]): A fixed-radius *probe* is rolled across the surface of a molecule, and the solvent-accessible surface (SAS) is the 2D surface traced-out by the center of the probe sphere. Typically fixed at 1.4 Å (water), larger probe radii are useful to create lower-resolution, coarse-grained images of molecular envelopes in large (>100 Å-scale) assemblies (*e.g.*, Fig. 3 in [28]). Note that surfaces most accurately reflect (cf. ribbons, sticks) the biophysical properties of the underlying molecule, and much of biochemical function (*e.g.*, protein···ligand interactions) occurs at the SAS. The well-studied correlations between SASA and physicochemical properties (protein-binding affinities, free energies of solvation) have led to useful heuristics, such as a linear relationship between buried surface area and free energy of binding, the slope being ≈ 20-30 cal mol^{-1} $Å^{-2}$. [29] Surface renditions are well-suited to illustrate such $\Delta G°$ ↔ SASA trends across a series of molecular complexes. Further information on surfaces (excluded, buried, occluded) and related geometric concepts (*contact surfaces*, *reentrant surfaces*) can be found in ref. [1] and in Connolly's review [30].

Beyond the standard molecular surface, some questions may require considering additional topographic and topological features of a structure – For instance, pockets and voids are internal regions depleted in

atomic density, and which may be located and characterized (surface area, volume, mean local curvature, *etc.*) *via* grid-based calculations. Visualization of such regions is of interest in drug design (potential ligand-binding pockets, atomic affinity grid maps [31]), membrane protein channels and transporters, physicochemical issues of protein structure and thermodynamics (differential hydration of internal voids in 'loose' versus tightly-packed structures), and numerous other areas. Similarly, 'trenches' may exist on the surface of a protein, and 'tunnels' or 'gorges' may lead to active sites or allosteric effector sites; efficient visualization of such regions is necessary in studying, for instance, the diffusional accessibility of an enzyme active site or permeability properties of a membrane transporter. The 'HOLE' software [32,33] is often used to compute, analyze, and visualize such pores and channels, and Nayal & Honig's recent study of protein cavities [34] provides many references to related topics and software packages.

The development of integrated tools for seamless visualization and quantitative querying of molecular properties is an active area of research. Quantitative properties, such as molecular areas and volumes, are often pre-computed and grafted into PDB files in an off-line, pre-processing stage – for instance, residue-based accessibilities can be mapped to *B*-factor or occupancy fields. However, the APIs of modern software packages, such as VMD, provide commands for readily computing such properties on-the-fly (*e.g.*, over frames of an MD trajectory) and flexibly handling the resultant output (map to atoms in the session, write to files, *etc.*). A recent development in the visual data-mining of MD simulations offers a structural query approach that is both flexible *and visual*: Sourina *et al.* [35] introduced a method wherein the user defines a simple geometric 'query region' (*e.g.*, a cylinder), within which sets of atoms and other properties of interest can be computed on-the-fly (*via*, *e.g.*, set theoretic operations such as *atoms_of_interest* = *molecule* ∩ *solid cylindrical region*, with the structure of both the *molecule* and the *query region* treated as free to dynamically evolve over the course of the trajectory).

2.6) 'Materials' and advanced concepts

The *material properties* of molecular objects dictate the appearance of a rendered image. Every object in a scene consists of graphical primitives – be it opaque or semi-transparent, atomistic (atoms, bonds) or not (backbone ribbon), a derived property (*e.g.*, surfaces) or otherwise (*e.g.*, electron density mesh). These primitives have adjustable material properties that literally define their interactions with impinging light rays, and therefore determine the final appearance of the primitives in a rendered scene. Material characteristics include the specular, diffuse, and ambient components of reflectivity; material color; and degree of transparency (defined by an alpha value from 0.0 (fully transparent) to 1.0 (opaque)). For instance, *matte* materials exhibit highly *diffuse* scattering, whereas a shiny material has significant *specular* reflectivity. VMD supplies many (customizable) materials for interactive rendering, with descriptive names such as 'BrushedMetal', 'HardPlastic', *etc.*; in PyMOL, '`ray_texture*`' settings modify material properties at the stage of ray-tracing.

The concept of a *material* is built upon in the method of "texture mapping", yielding objects with more realistic (or otherwise intricate) appearances. Invented by Blinn [36], Catmull [37], and coworkers in the 1970s, texture mapping has been utilized in MolVis [17,18,38,39,40,41] and other application domains. In this technique, a 2D 'texture' – a bitmap of texture elements ('*texels*'), often of greater simplicity than the target object – essentially serves as a decal that is *mapped* onto a spatial domain, such as a 2D molecular surface in 3D space. This approach to defining a surface (*versus*, *e.g.*, tessellating with triangles) affords many computational advantages and possibilities for scene construction, including smooth real-time rendering of complex shapes featuring high local curvature, and efficient visualization of large volumetric datasets (*e.g.*, electrostatic maps) *via* 3D texture mapping [42,43]. Extensions of the texture map idea, such as *normal mapping* [38], enable the vis pipeline to achieve the accuracy and realism of sophisticated lighting and shading models (*e.g.*, Phong shading versus the simpler Gouraud), in a computationally feasible manner for complex shapes such as proteins. Also, texture mapping has been efficiently combined with the marching cubes surface extraction algorithm to enable efficient isosurface generation and real-time modification (*e.g.*, recontouring of electron density maps) [38]. Finally, while a 2D-decal-onto-3D-object is relatively intuitive, note that the texture mapping concept is entirely general: Textures of arbitrary dimensionality may be mapped (parametrically or otherwise) onto any well-defined

domain. For instance, 1D textures (color-maps, opacity gradients, *etc.*) can be applied onto 2D or 3D solid objects, and feasible real-time 3D texture mapping has been achieved *via* wavelet-based encoding of solid (3D) textures [42]. A somewhat related concept is that of 'impostor' rendering, wherein simple entities (2D projections of cylinders [bonds], spheres [atoms], *etc.*) are taken as visual hulls that can be parametrically mapped(/textured) onto the 2D rectangular quad of the viewing plane for the molecular objects. The TexMol software of Bajaj et al. implements *textural imposters* [43] and, as a potentially more efficient alternative to triangular tessellation for surface rendering, Tarini *et al.* recently integrated *procedural impostors* with advanced methods for texture mapping, ambient occlusion lighting, and edge-enhanced cueing to achieve a new, stunningly effective approach to molecular visualization (see [12] and references therein). Other recent developments include new texture map-based methods for treatment of subsurface scattering (important for translucent materials; [44]), as well as more computationally flexible handling of textured surfaces (*e.g.*, allowing deformability) *via* a "decal compositing" approach [45]. It is likely only a matter of time before these new computer graphics and visualization approaches begin to be adopted in the MolVis software community.

2.7) Beyond flatland: Stereoscopic viewing

We live in a three-dimensional world, meaning our effectively planar retinas 'see' only 2D projections of 3D objects [46]. But, with two laterally-offset eyes, our visual cortex can integrate binocular vision and past visual experience to reconstruct a visual *perception* of the 3D object being viewed. These principles are utilized in MolVis to enhance depth perception *via* stereoscopic viewing. Molecular representations can be drawn, in print or on-screen, in a monocular (single-eyed) mode, or as a stereoscopic (binocular) pair. Stereo-pairs can be generated and viewed in different ways. For instance, distinct left- and right-eye images can be drawn for manual viewing. One achieves the 3D effect either *(i)* unaided, by relaxing eye muscles to view the image directly ('divergent' or 'wall-eyed' stereo) or by crossing one's eyes ('cross-eyed' stereo); or *(ii) via* spectacles featuring a mirror system that enforces the correct matching between left/right image ↔ left/right eye. Such '*side-by-side*' methods are characterized by the *stereo divergence* between the images (generally ≈ 5°). For on-screen viewing, sophisticated hardware such as stereo-shutter glasses can be used. The basic principle of these (now-affordable) hardware technologies is that the graphics card toggles between left- and right-eye images, such that alternation between images *(i)* is synchronized with the alternation in left/right panels (shutters) of the user's stereo-glasses, and *(ii)* occurs at sufficiently high frequency to avoid visible flickering (thereby demanding screen refresh rates > 120 Hz). Though somewhat dated in terms of hardware capabilities and availabilities, ref. [47] provides a useful account of stereoscopic software and hardware issues.

2.8) Curves, splines, and interpolation

Curves known as '*splines*' are ubiquitous in MolVis, but these geometric objects involve lingo that may be relatively unfamiliar. The basic idea of splines is that of interpolation: Use curves to 'fill-in' a set of points (*e.g.*, C_α positions) by approximating the path of the point-set using simple functional forms (linear, polynomial, *etc.*). This is essentially a form of curve-fitting, subject to the constraint that the fitted curve contains the data points (or "control points", in the language of splines). The PyMOL setting '`cartoon_nucleic_acid_mode`' can be adjusted to experiment with this behavior.

Linear interpolation is a simple form of approximation. Often used to 'morph' between states A and B of a biopolymer (S) *via* a series of discrete structural intermediates, linear interpolation between $S_A \rightarrow \cdots S_i \cdots \rightarrow S_B$ can be used to generate S_i frames to animate $A \leftrightarrow B$ conformational transitions, create motion-blur static images, and so on; note that the two structural extrema (A, B) are the only two real 'data' points in this example. Accurate fitting of complex shapes, such as a constellation of C_α points, requires the more sophisticated approaches of nonlinear interpolation. For instance, splines are interpolated curves constructed piecewise across intervals, *via* polynomial functions that are fit smoothly together; hence, *e.g.*, the oft-encountered 'cubic spline' refers to polynomials of degree three. Constructing splines as linear combinations of different families of basis splines (B-splines) generalizes and unifies these ideas, including the Bézier curves used in image post-processing programs such as *Illustrator* (Box S1).

Finally, note that the most routine use of splines may go unnoticed: Cartoon and ribbon diagrams are the predominant rendering style in MolVis, and are computed by fitting splines to a protein backbone (or any other atom selection). The properties of these splines are accessible at a lower-level than one might suspect. For instance, VMD's 'NewRibbons' and 'NewCartoon' reps offer pull-down menus that allow one to toggle between *B*-spline and Catmull-Rom; the latter is a variety of cubic (Hermite) spline used in computer graphics because of its accuracy and numerical efficiency.

2.9) Animations

Molecular animations are useful in many contexts, and for many reasons. Chief among these is the fact that animations simplify complex datasets, such as the 3D structure of a biopolymer, by distributing the data across an additional dimension – time. For instance, a protein–ligand complex can be gradually rotated by 180° over the course of a movie to reveal the otherwise hidden (and potentially informative) regions of a putative binding site, and to enhance one's sense of depth perception *via* the "kinetic depth effect" [48]. Also, animations are the most natural means for visual analysis of MD simulations, as these trajectories are a time-series of 3D structural snapshots.

Animations can be systematically classified into four basic types (Table S1), depending on what is to be shown (*e.g.*, a static structure *versus* MD trajectory) as well as the exact aim of the particular animation – *e.g.*, a comprehensive scan of the entire surface of a protein complex (requires many perspectives), *versus* monitoring conformational changes from a fixed viewpoint (single perspective). Thus, animations can be categorized based on whether the molecular object is static (M_s) or dynamic (M_d), and whether the viewpoint is fixed (V_s) or dynamically changing (V_d), as shown in Table S1. M_sV_s corresponds to a 'regular' (static) figure rather than a typical (moving) animation. M_sV_d corresponds to a static molecular object viewed from dynamically changing perspectives, the main purpose of this type of animation being to view multiple parts of the molecule (*e.g.*, regions of an active site that may be obscured at the beginning of the animation, but become clearly visible by the end), or with different representation styles (CPK, ribbon, *etc.*) fading on/off at different stages in the animation; as M_sV_d corresponds to rigid-body rotation and translation with possible scaling/zooming, it can be expressed mathematically as an affine transformation. Animations of a dynamically fluctuating molecule from either static (M_dV_s) or dynamic (M_dV_d) viewpoints reveal changes in molecular coordinates over a series of frames. Variations in coordinates (M_d) *versus* frame number can be *(i)* purely geometric, as in linear interpolation between structural endpoints 'A' and 'B' (in which case frame number corresponds to morphing progress); or *(ii)* from a physical (force field-based) simulation, such as in MD trajectories (where frame number corresponds to straightforward passage of time).

The boundaries between the categories in Table 1 are not absolute, and can be blurred in potentially useful ways. For instance, consider M_sV_s and M_dV_s: a 'motion-blur' representation style may be a highly versatile graphical device for effectively conveying many different types of 'dynamical' information, including *(i)* conformational heterogeneity inferred from individual (static) crystal structures; *(ii)* structural variability in an NMR ensemble; *(iii)* interpolation between two structural endpoints; *(iv)* projections of structures along eigenvectors from a normal mode analysis; and *(v)* structural snapshots along time-slices of an MD trajectory. This increasingly popular technique essentially corresponds to the film industry's concept of "onion skinning" or "tweening" (this being the term from the CGI field) as a way of achieving motion-blur effects. An example of this type of graphical device can be found in, for instance, illustrations of Ras conformational dynamics (Fig. 2 of ref. [49]).

2.10) Post-processing, Vector and raster graphics

One of the most fundamental concepts in MolVis surrounds the terms '*vector*' and '*raster*' ('*bitmap*' and '*framebuffer*' are used synonymously with *raster* for reasons explained in [1]). The basic idea (Box 1) is used in reference to at least two distinct sets of issues: *(i)* a hardware display (and the low-level algorithmic implementations to drive it) and *(ii)* image file formats and desktop publishing software. As alluded to above (§1.2), usage of vector graphics hardware waned because of limitations in this method of displaying images on the screen *via* monochrome lines, polygons, *etc.* drawn by a CRT gun; though computationally efficient because of the simple (analytical) representation of graphical entities as vectors,

the vector method is not as amenable to complex effects, such as color, lighting, texturing, etc. In contrast, raster graphics readily enable implementation of advanced graphical methods – colorization (a 24-bit pixel can display 16M colors); sophisticated shading and lighting models; alpha-blending for multi-layer, multi-color transparency; photorealistic rendering *via* methods like ray-tracing, and so on. Users generally encounter this issue in the post-processing stages.

3) Abbreviations used in this supplementary text

3D, three-dimensional; CPK, Corey-Pauling-Koltun; GPU, graphics processing unit; MD, molecular dynamics; PDB, Protein Data Bank; rep, representation; RMSD, root-mean-square deviation; SA, surface area (as in SASA for "solvent accessible surface area"); SSE, secondary structure element; SVD, singular value decomposition; vdW, van der Waals; VMD, Visual Molecular Dynamics software; vis, visualization (as in MolVis for "molecular visualization")

Box S1: Practical tips for molecular illustrations

Tip 1: Articulate the goals of the image, select representations accordingly. This entails scientific and non-scientific questions. What is the exact scientific goal of the image? What length-scale do the molecular objects occupy (Fig. 1)? This will dictate the choice between atomistic (wireframe, stick), backbone-level (trace, ribbon cartoon), or highly schematic reps (*e.g.*, molecular envelope), as well as whether the ideal rendition will be surface, space-filling, volumetric, *etc*. Also consider alternatives such as mono/stereo figures, projection/orthographic rendering, and so on. A less scientific, but equally important, consideration is final figure geometry (dimensions and page-layout). For instance, graphics are often assembled into multi-panel figures that span 2-3 columns of a page; a molecular region such as an active site may be displayed as surfaces in one panel and all-atom sticks in the abutting one. Such decisions are most conveniently made early-on in creating scenes.

Tip 2: Optimize scenes. In practical terms, this is the crux of the figure-creation process. Once the scientific goal has been set, a scene is constructed by loading the relevant molecules into a MolVis session, defining atom selections, adjusting representations, hidings sets of atoms, drawing lines to indicate key distances, *etc*. The scene can be tuned *via* literally hundreds of settings; those properties most strongly influencing a scene's appearance are discussed in the text (*e.g.*, lighting/shading, depth-cueing). Experiment with many settings to obtain an 'optimal' scene that fulfills the original scientific goal in a manner that is accurate, clear, and aesthetically appealing.

Tip 3: Get your bearings. Visually study the molecules early in the process of creating a scene, and settle upon a few useful orientations. Multiple 3D perspectives likely will be necessary to show different molecular features but, to avoid confusion, *(i)* limit the number of views and zoom levels, and *(ii)* use symbols, arrows, and other diagrammatic conventions to clearly indicate the relationship of alternate views to the initial 'canonical' orientation.

Tip 4: Accentuate important features. Highlight the most relevant molecular regions in an image, either during post-processing (*e.g.*, text labels on important residues) or via more indirect means during raw image construction (*e.g.*, carefully-tweaked depth-cueing, hiding unimportant or irrelevant features *via* 'fog' or subdued coloring).

Tip 5: Complexity & simplicity. The best molecular graphics are a compromise between complexity and simplicity, as implied by Fig. 1. As an example of extreme complexity, consider that it is possible to show *six* dimensions of data in a single image of the often-studied (and illustrated) DNA duplex known as the Dickerson dodecamer. How? There are *(i)* three spatial coordinates of atoms; *(ii)* atomistic properties (*e.g.*, *B*-factors) can be mapped onto the structure (*e.g.*, as a color gradient; at the level of atoms, ribbons, surfaces); *(iii)* because, in this special case, the DNA sequence is self-complementary, one 'half' of the molecule could be used to display one set of properties (of the sort listed in *(ii)*), and the remainder can be used to show another set of properties (*e.g.*, electrostatic potential on one half, counterion densities on the other half); and *(iv)* this already-complex representation, incorporating tricks *(i)→(iii)*, can be rotated in time, thereby introducing yet another dimension of variability (dynamics). Even when viewed stereoscopically, such a complex representation is likely to be *incomprehensible*. At the other extreme, over-simplification leads to a figure that is thin in information, and which likely should be replaced by a simple sentence of text; for instance, depending on the readership, it is unlikely to be useful to show a default ribbon diagram of the Dickerson dodecamer, as thousands of such representations already exist in the literature.

Tip 6: Colors. Related to Tip 4, color can be effectively used to accentuate salient features of an illustration. But begin by asking if color is absolutely essential. If so, use color sparingly, in a meaningful way. For instance, strive to limit the number of colors – too many colors leads to difficulty or outright confusion in comprehending a figure. Be consistent with color schemes, and utilize graphical conventions to aid the viewer. For instance, match the colors of text labels to the objects they refer to. Note that color also can be used in less standard ways; *e.g.*, to indicate progress along a time-slice in MD trajectories. Do not overlook the color of the background (the 'canvas' in many MolVis programs); it is generally good practice to use a white background,

as that is more compatible with photocopying, wastes less printer ink, and permits a less saturated color space to get the same level of contrast as on a dark background. Finally, note that papers containing color figures are often printed on black/white printers; a good practice is to print a newly-created piece of graphics to verify that it is comprehensible in grayscale. For example, the red/blue extremes of the common "heat maps" appear identical when converted to grayscale. (A simple print test also helps address issues of image appearance in RGB (monitor) *versus* CMYK (printing) color-spaces.)

Tip 7: Transparency. Spheres and surfaces are the representations most often rendered semi-transparently. While it can be used effectively, note that poorly designed / implemented transparency results in unclear and even confusing images. In low-level (OpenGL) terms, opacity is a material property known as 'alpha', ranging from 0 (perfectly transparent) to 1.0 (completely opaque); for instance, a 'transparent' object defaults to α=0.3 in VMD. A frequent temptation is to use many levels of opacity – *e.g.*, an α=0.2 surface, underlying ribbons at α=0.7, and active site atoms at α=1. However, maintaining visual clarity at this level of complexity is almost impossible; experiment with PyMOL's 17 transparency-related settings, adjust its ray-tracer settings ('ray_trace_mode'), and so on. Also, multi-layer (alpha-blending) settings can be adjusted to make semi-transparent objects clearer in a final image. Note that advanced techniques like multi-layer transparency will make ray tracing far more computationally costly; with large objects such as the ribosome, these effects will tax even the most expensive / high-performance graphics cards.

Tip 8: Learn scripting by reverse engineering. An efficient way to advance from novice to expert is to use a MolVis package's logging functionality to reverse-engineer desirable behavior (*e.g.*, a particularly nice scene that you constructed manually). This approach is effective because it exposes *exactly* what commands and settings led to *exactly* what graphical output. More explicitly, suppose that a series of manipulations was used to set-up a desired scene ready for rendering into a publication image (load PDB → select atoms → show as CPK → ···· → ray-trace). It is generally true, particularly for beginners, that the sequence of scene-construction steps was performed manually, using the GUI rather than the software's command line / API to issue commands, execute macros, tweak settings, *etc*. A basic feature of modern MolVis programs is that any scene created *via* manual/interactive usage can also be expressed as a sequence of (text-based) commands and settings – *i.e.*, a 'script'. The software's logging functionality can capture the manually-executed steps to a plain-text logfile. This logfile provides a mechanism for reverse-engineering what was done, thereby teaching you the effects of all the commands and settings. Both PyMOL ('`log_open`') and VMD ('`logfile`') offer commands to activate such 'logging' modes.

Tip 9: Post-processing: Raw images → published figure. For simplicity (and therefore robustness), minimize the amount of necessary post-processing by incorporating into the graphical scene, before rendering into a final image file, as much of the intended imagery as possible (labels for key residues, N'-/C'-termini, *etc.*). Tasks which are not well-supported by MolVis programs – *e.g.*, adding labels to β-strands at specific positions along each strand – are best performed during post-processing in a desktop publishing / graphics editing suite. For instance, a useful tactic to make b-strand labels visible in complex backgrounds is to apply an "outward glow" to the text. Such stylistic effects can be achieved in many programs, including Adobe's *Photoshop* and *Illustrator*, as well as open-source alternatives such as the *GNU Image Manipulation Program* (*GIMP*) or *Inkscape*.

Tip 10: Vector versus raster. Vector-based tools (*Illustrator*, *Inkscape*) may be preferred over raster/bitmap (*GIMP*, *Photoshop*) because vector images can be magnified without loss of resolution, whereas the appearance of a bitmap significantly degrades upon zooming ('pixelization'); this is because vector graphics represent images *via* geometric primitives (lines, curves(/splines), polygons, *etc.*) that are arbitrarily scalable, whereas raster/bitmap graphics utilize a (fixed) array of pixels. The terms 'vector' and 'bitmap/raster' also refer to image file formats, examples of vector formats being SVG, Postscript, and Adobe Illustrator files, while popular bitmap formats include JPEG, PNG, and TIFF. One can interconvert vector ⇆ raster formats, although the reverse direction (vectorization) is imperfect. For instance, on a Linux workstation with the ImageMagick software, the command-line "`convert -density 300 vector_file bitmap_file`" would convert (specifically, 'rasterize') the vector-formatted input file into a bitmap file at 300 dots/inch (the resolution used by most publishing houses for color artwork).

Supplementary Figure Captions, Video Descriptions

Supporting figures S2→4 utilize 3D structural data from the following four biomolecular systems (the first three of which are interrelated): *(i)* a high-resolution crystal structure of the bacterial Sm-like protein 'Hfq', which forms hexamers that bind RNA [50]; *(ii)* the crystal structure of an Sm-like archaeal protein tetradecamer from *Pyrobaculum aerophilum* ('*Pae* SmAP3'), determined to a resolution of 2.0 Å [51]; *(iii)* the recent structure of *E. coli* Hfq co-crystallized with a polyriboadenylate 15-mer RNA (poly(A); 'A_{15}'), determined to 2.4 Å resolution [52]; and *(iv)* the crystal structure of a dUTPase from *Mycobacterium tuberculosis* [53]. Figure S2 utilizes the first system (Hfq) to demonstrate the variety of possible representation styles, applied in the context of a potentially difficult (oligomeric) system. Figure S3 employs the second system (SmAP3) to illustrate an even higher-order (14-mer) assembly, as well as high-resolution structural details such as an ion-binding site. Finally, figure S4 demonstrates the potential ways in which volumetric data can be represented, such as in the frequently-encountered renditions of electron density (dUTPase) or electrostatic potentials (Hfq); PyMOL-based calculation and visualization of electrostatic potentials are illustrated for the Hfq•A_{15} system.

Figure S1. Biomolecular graphics in a nutshell. In serving as a roadmap for the figures (inset labels), this diagram illustrates the principle that, to create biomolecular graphics which are both comprehensible and meaningful, one must carefully balance the level of representation detail (ordinate) against the complexity of the molecular entities (atoms, molecules, *etc.*) to be illustrated (abscissa). Because our ability to visually comprehend a graphical scene is limited, representation granularity and object complexity are counteracting forces, and the curve slopes downward. The hypothetical upward concavity at long length scales suggests the possibility that a 'saturation point' is reached at some critical level of complexity. For instance, one cannot draw hundreds of unit cells of a protein crystal while retaining the representation of the object of interest as a discernable protein (*versus*, say, a schematic icon); this effect asymmetrically influences the curve because, in the limit of small length scale, one reaches the atom. The thickness of the curve is to imply that there is no 'magic formula', but rather a range of probably-lucid representations. Finally, as one deviates perpendicularly away from the curve, the increasing difficulty of representing particular combinations of length scales and representation styles can be considered as a sort of "representation pressure".

Figure S2. Different representation styles and their relative utility. Panels A → F illustrate the high-resolution (1.55 Å) X-ray crystal structure of *Staphylococcus aureus* Hfq hexamer [50]. The representation styles which are shown include (A) backbone (C_α) trace; (B) smooth backbone 'tubes' (splines); (C) a stick-like representation (technically, a 'ball-and-stick' rep, with the ball/stick ratio scaled as specified in the PyMOL script); (D) a classic ribbon cartoon, wherein β-strand directions are indicated as arrows; (E) space-filling CPK spheres (the "ambient occlusion" effect is achieved *via* lighting methods provided in the PyMOL script); and (F) a molecular surface overlaying the cartoon representation of panel (C). For clarity, an individual Hfq monomer is delimited by dashed lines in (D), and strands are labeled consecutively from the N′ → C′ termini (a neighboring subunit is distinguished by primes). A variable-width 'putty' representation is shown in (G) for one of the 14-mers from the asymmetric unit of that SmAP3 crystal structure [51]. In this representation style, the diameter of the backbone spline is scaled by the value of some (user-specified) atomic property that varies along the chain (in this case, *B*-factor). The power of this illustration is that it clearly shows that elevated *B*-factors (presumably reflecting static or dynamic disorder) occur along the periphery of the large complex. Also notice the particular conventions that were used to aid visual interpretation of this graphic. For instance, *(i)* in panels A–F, saturation intensity of the blue color alternates from one subunit to another, making the hexameric layout of Hfq readily apparent from just a glance; *(ii)* in panel (G), various graphical devices and diagrammatic symbols are used to indicate important structural regions, relative orientations in different images in the sub-panels, and so on.

Figure S3. A tetradecamer assembly; Overall architecture, domain organization, and ion-binding. The SmAP3 14-mer of Fig. S1G is further illustrated in these renditions. As shown in (A), ribbon cartoons that utilize a carefully-chosen color scheme can convey information about both the fold of the SmAP3 monomer, as well as the overall organization of subunits in terms of two SmAP3 heptamers (*apical* and *equatorial*). The two rings – in blue (equatorial) and orange (apical) hues – stack upon one another in a *head–head* manner, but adopt distinct conformations (hence the different hues). Within each ring, an N-terminal domain (NTD) of Sm proteins (orange[api] / blue[equ]) is augmented by C-terminal domains (CTD) that are analogously colored (light orange / light blue). A single subunit of the 14-mer is accentuated in red–yellow, where red corresponds to the NTD_{Sm}, and the CTD is yellow. Novel Cd^{2+}–binding sites are shown as green spheres, and at successively higher resolution in panels (B) and (C). A rectangular box in (B) indicates the 'zoomed-in' region of panel (C). Labels are used to clearly identify relevant molecular entities, such as the amino acid side-chains and water ligands that chelate Cd^{2+} in a distorted tetrahedral geometry (C).

Figure S4. Volumetric data. Volumetric quantities, such as electron density or electrostatic potentials, are often the most challenging type of data to illustrate effectively. Chief among the reasons for this is the fact that such data are continuous through 3D space, rather than being discretely 'chunked' into atoms, bonds, residues, *etc.*; thus, volumetric data (which generally may be scalar- or vector-valued) are not amenable to representation using the well-established graphical devices of vertices (atoms), lines (bonds), splines (backbone cartoon), triangles (surfaces), and so on. Conventional rendition styles include the isocontour "chicken-wire" meshes that are ubiquitous in crystallography. For instance, an electron density "omit map" at the active site of a *Mycobacterial* dUTPase is shown in (A); note the use of text labels and dashed lines to indicate important structural features of this enzyme. Panels B→E illustrate additional approaches for volumetric data; the utility of each method depends strongly on the specific question addressed by the graphic. For instance, if the aim is to convey information about the electrostatic properties of a molecule, surfaces can be drawn at constant values of the electrostatic potential; these "electrostatic surfaces" are often shown as positive / negative pairs, such as the +3 (blue) and –3 (red) $k_B T/e$ isocontours shown in (B). However, such images are often quite cluttered, and are therefore of somewhat limited utility. Less 'busy' images generally can be attained by mapping the values of the potential onto a surface, such as the solvent-accessible surface or the molecular (Connolly) surface shown in (C). In this example, note the steric *and* electrostatic complementarity between single-stranded poly(A) RNA (sticks) and the surface of the Hfq hexamer. Perhaps the least traditional representation approaches are *(i)* to directly visualize the field lines corresponding to the gradient of electrostatic potential, as in (D), or *(ii)* to take a 2D 'slice' through 3D volumetric data, as in (E). Volumetric vector fields are often visualized as illuminated streamlines [54]; advanced methods for computation and visualization of such 3D vector fields are beyond the scope of this guide, but recent developments can be found in [55].

The structure of an *E. coli* Hfq•poly(A) complex was used for the electrostatics calculations illustrated in this figure and in video 2; specific details can be found in the accompanying PyMOL scripts and in the Supplementary Video 2 'HowTo' guide.

Video S1. **DNA helicase.** This animation shows the conformational changes in a helicase as it unwinds double-stranded DNA. The movie is of type M_dV_s (using the nomenclature of Table1), and was produced in animated GIF format using PyMOL and the scripts accompanying this primer.

Video S2. **Electrostatics.** This screencast video is a step-by-step demonstration of the usage of PyMOL's APBS plugin to seamlessly integrate *(i)* the set-up and execution of a Poisson-Boltzmann electrostatics calculation with *(ii)* visualization of the resulting grid maps. The steps were performed on a GNU/Linux workstation, using relatively recent releases of the APBS (v1.1) and PyMOL (v1.2) packages.

Supplementary Tables

Table S1: A classification scheme for illustrations and animations.

		Coordinates of molecular entities (M)	
		static	***dynamic***
Viewpoint (V)	***static***	M_sV_s<br>*A standard (static) molecular illustration*	M_dV_s<br>*Visualize an MD trajectory from a single/fixed viewpoint*
	dynamic	M_sV_d<br>*A rotating camera 'scan' of the molecular objects*	M_dV_d<br>*Visualize a trajectory from changing viewpoints*

Possible types of 3D figures and animations are enumerated in this table, and discussed in the text. Below each symbol is a tangible example of that particular type of illustration. The principles and practices used to create static figures (M_sV_s) also apply to the design and rendering of animations, each frame of which is essentially a stand-alone figure.

Supplementary References

1. Bottomley S, Helmerhorst E (2009) Molecular Visualization. In: Gu J, Bourne PE, editors. Structural Bioinformatics. 2nd ed. Hoboken, NJ: John Wiley & Sons.
2. Dickerson RE (2009) Chapter 2: myoglobin: a whale of a structure! J Mol Biol 392: 10-23.
3. Dickerson RE (2005) Present at the Flood: How Structural Molecular Biology Came About. Sunderland, MA: Sinauer Associates, Inc. 307 p.
4. Gillet A, Sanner M, Stoffler D, Olson A (2005) Tangible Interfaces for Structural Molecular Biology. Structure 13: 483-491.
5. Jones TA (1978) A graphics model building and refinement system for macromolecules. Journal of Applied Crystallography 11: 268-272.
6. Editorial (1997) String and sealing wax. Nat Struct Biol 4: 961-964.
7. Levinthal C (1966) Molecular model-building by computer. Scientific American 214: 42-52.
8. Sayle RA, Milner-White EJ (1995) RASMOL: biomolecular graphics for all. Trends Biochem Sci 20: 374.
9. Whitted T (1980) An improved illumination model for shaded display. Commun ACM 23: 343-349.
10. Palmer TC, Hausheer FH, Saxe JD (1989) Applications of ray tracing in molecular graphics. Journal of Molecular Graphics 7: 160-164.
11. Merritt EA, Murphy MEP (1994) Raster3D Version 2.0. A program for photorealistic molecular graphics. Acta Crystallographica Section D Biological Crystallography 50: 869-873.
12. Tarini M, Cignoni P, Montani C (2006) Ambient Occlusion and Edge Cueing for Enhancing Real Time Molecular Visualization. Visualization and Computer Graphics, IEEE Transactions on 12: 1237-1244.
13. Stone J (2009) Tachyon: Parallel / Multiprocessor Ray Tracing System. http://jedi.ks.uiuc.edu/~johns/raytracer
14. Dickerson RE (1997) Obituary: Irving Geis, 1908-1997. Structure 5: 1247-1249.
15. HHMI (2000) Howard Hughes Medical Institute Purchases Geis Archives. http://www.hhmi.org/news/geis.html
16. Humphrey W, Dalke A, Schulten K (1996) VMD: visual molecular dynamics. J Mol Graph 14: 33-38, 27-38.
17. Olson AJ (2006) Molecular graphics and animation. In: Rossmann MG, Arnold E, Fuess H, Hahn T, Wondratschek H et al., editors. International Tables for Crystallography. Chester, England: International Union of Crystallography.
18. Goddard TD, Ferrin TE (2007) Visualization software for molecular assemblies. Curr Opin Struct Biol 17: 587-595.
19. Martz E, Kramer T (2006) World Index of Molecular Visualization Resources. http://molvisindex.org
20. Martz E, Francouer E (2004) Online History of Molecular Visualization. http://www.umass.edu/microbio/rasmol/history.htm
21. Kautz J (2004) Hardware lighting and shading: a survey. Computer Graphics Forum 23: 85-112.
22. Goodsell DS (2005) Visual Methods from Atoms to Cells. Structure 13: 347-354.
23. Dickerson, R E, Geis I (1969) The structure and action of proteins. Menlo Park, CA: W.A. Benjamin.
24. Richardson JS (2000) Early ribbon drawings of proteins. Nat Struct Biol 7: 624-625.
25. Lawrence MC, Colman PM (1993) Shape complementarity at protein/protein interfaces. J Mol Biol 234: 946-950.
26. Lee B, Richards FM (1971) The interpretation of protein structures: Estimation of static accessibility. Journal of Molecular Biology 55: 379-IN374-379-IN374.
27. Shrake A, Rupley JA (1973) Environment and exposure to solvent of protein atoms. Lysozyme and insulin. J Mol Biol 79: 351-371.
28. Arluison V, Mura C, Guzman MR, Liquier J, Pellegrini O, et al. (2006) Three-dimensional structures of fibrillar Sm proteins: Hfq and other Sm-like proteins. J Mol Biol 356: 86-96.
29. Janin J, Bahadur RP, Chakrabarti P (2008) Protein-protein interaction and quaternary structure. Q Rev Biophys 41: 133-180.

30. Connolly ML (1996) Molecular Surfaces: A Review. http://www.netsci.org/Science/Compchem/feature14.html
31. Goodford PJ (1985) A computational procedure for determining energetically favorable binding sites on biologically important macromolecules. J Med Chem 28: 849-857.
32. Smart OS, Neduvelil JG, Wang X, Wallace BA, Sansom MS (1996) HOLE: a program for the analysis of the pore dimensions of ion channel structural models. J Mol Graph 14: 354-360, 376.
33. Goodfellow JM, Pitt WR, Smart OS, Williams MA (1995) New methods for the analysis of the protein-solvent interface. Computer Physics Communications 91: 321-329.
34. Nayal M, Honig B (2006) On the nature of cavities on protein surfaces: application to the identification of drug-binding sites. Proteins 63: 892-906.
35. Sourina O, Korolev N. Visual Query Interface for Molecular Dynamics Application; 2009. IEEE Computer Society. pp. 298-304.
36. Blinn JF, Newell ME (1976) Texture and reflection in computer generated images. Commun ACM 19: 542-547.
37. Catmull E (1974) A subdivision algorithm for computer display of curved surfaces [PhD]. Salt Lake City: University of Utah.
38. Teschner M, Henn C, Vollhardt H, Reiling S, Brickmann J (1994) Texture mapping: a new tool for molecular graphics. J Mol Graph 12: 98-105.
39. Duncan BS, Olson AJ (1995) Texture mapping parametric molecular surfaces. J Mol Graph 13: 258-264.
40. Weber JR (2009) ProteinShader: illustrative rendering of macromolecules. BMC Struct Biol 9: 19.
41. Henn C, Teschner M, Engel A, Aebi U (1996) Real-time isocontouring and texture mapping meet new challenges in interactive molecular graphics applications. J Struct Biol 116: 86-92.
42. Bajaj C, Ihm I, Park S (2000) Compression-Based 3D Texture Mapping for Real-Time Rendering. Graphical Models 62: 391-410.
43. Bajaj C, Djeu P, Siddavanahalli V, Thane A. TexMol: Interactive Visual Exploration of Large Flexible Multi-Component Molecular Complexes; 2004. IEEE Computer Society. pp. 243-250.
44. François G, Pattanaik S, Bouatouch K, Breton G (2008) Subsurface Texture Mapping. IEEE Comput Graph Appl 28: 34-42.
45. Schmidt R, Grimm C, Wyvill B. Interactive decal compositing with discrete exponential maps; 2006; Boston, Massachusetts. ACM. pp. 605-613.
46. Abbott EA (1884) Flatland: A Romance of Many Dimensions. United Kingdom: Seely & Co.
47. Rodriguez R, Chinea G, Lopez N, Vriend G (1999) Full window stereo. Journal of Molecular Graphics and Modelling 17: 310-314.
48. Wallach H, O'Connell DN (1953) The kinetic depth effect. J Exp Psychol 45: 205-217.
49. Gorfe AA, Grant BJ, McCammon JA (2008) Mapping the nucleotide and isoform-dependent structural and dynamical features of Ras proteins. Structure 16: 885-896.
50. Schumacher MA, Pearson RF, Moller T, Valentin-Hansen P, Brennan RG (2002) Structures of the pleiotropic translational regulator Hfq and an Hfq-RNA complex: a bacterial Sm-like protein. EMBO J 21: 3546-3556.
51. Mura C, Phillips M, Kozhukhovsky A, Eisenberg D (2003) Structure and assembly of an augmented Sm-like archaeal protein 14-mer. Proc Natl Acad Sci U S A 100: 4539-4544.
52. Link TM, Valentin-Hansen P, Brennan RG (2009) Structure of Escherichia coli Hfq bound to polyriboadenylate RNA. Proc Natl Acad Sci U S A 106: 19292-19297.
53. Chan S, Segelke B, Lekin T, Krupka H, Cho US, et al. (2004) Crystal structure of the Mycobacterium tuberculosis dUTPase: insights into the catalytic mechanism. J Mol Biol 341: 503-517.
54. Zöckler M, Stalling D, Hege H-C. Interactive visualization of 3D-vector fields using illuminated stream lines; 1996; San Francisco, California, United States. IEEE Computer Society Press. pp. 107-ff.-107-ff.
55. Helgeland A, Andreassen O (2004) Visualization of vector fields using seed LIC and volume rendering. IEEE Transactions on Visualization and Computer Graphics 10: 673-682.
