# Supplementary figures and images for: An Introduction to Biomolecular Graphics
Source: PLoS Comput Biol. 2010 Aug 26;6(8):e1000918. doi: 10.1371/journal.pcbi.1000918 (PMC2928806; doi:10.1371/journal.pcbi.1000918)

**Figure S1. Biomolecular graphics in a nutshell.**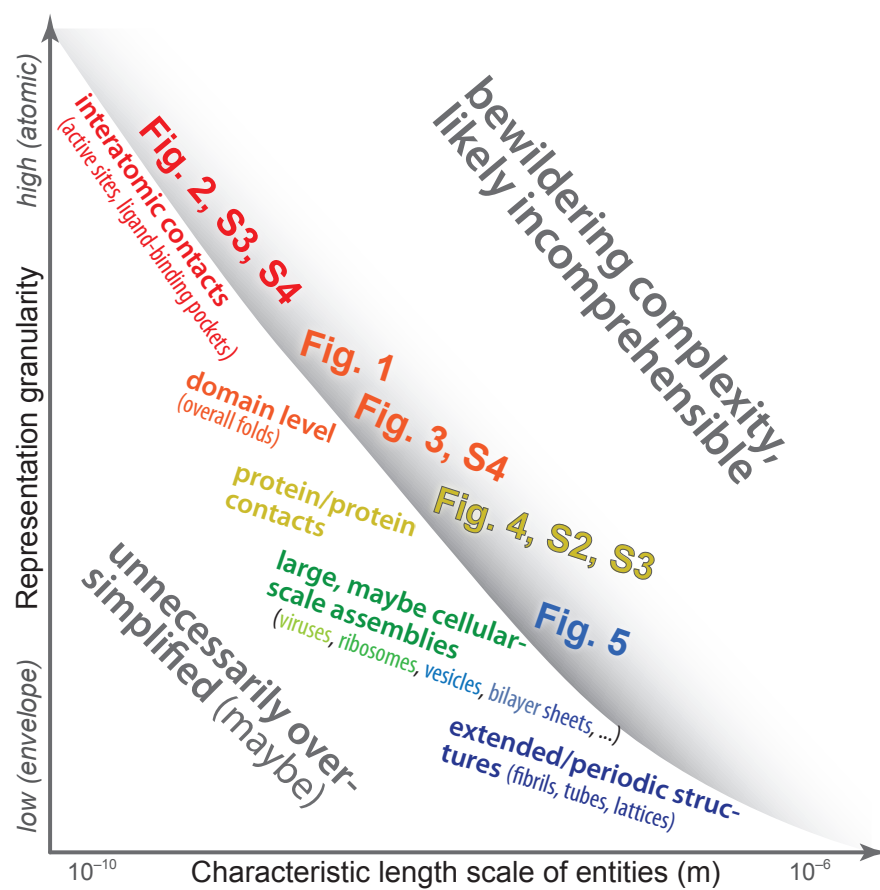

Supplement: Figure S1 — Biomolecular graphics in a nutshell. (0.82 MB PDF) [file pcbi.1000918.s002.pdf]

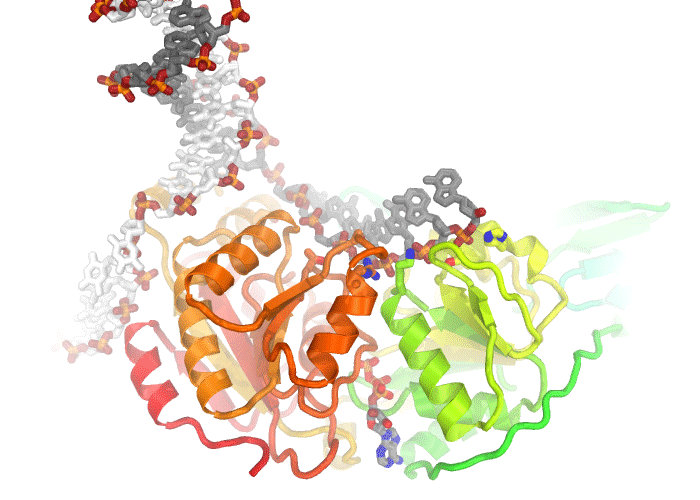

Supplement: Video S1 — Helicase animation. This animation shows the conformational changes in a helicase as it unwinds double-stranded DNA. The movie is of type MdVs (using the nomenclature of Table S1), and was produced in animated GIF format using PyMOL and the scripts accompanying this primer. (2.22 MB GIF) [file pcbi.1000918.s006.gif]
