# Supplementary material for: An Introduction to Biomolecular Graphics
Source: PLoS Comput Biol. 2010 Aug 26;6(8):e1000918. doi: 10.1371/journal.pcbi.1000918 (PMC2928806; doi:10.1371/journal.pcbi.1000918)

Figure S2. Different representation styles and their relative utility.

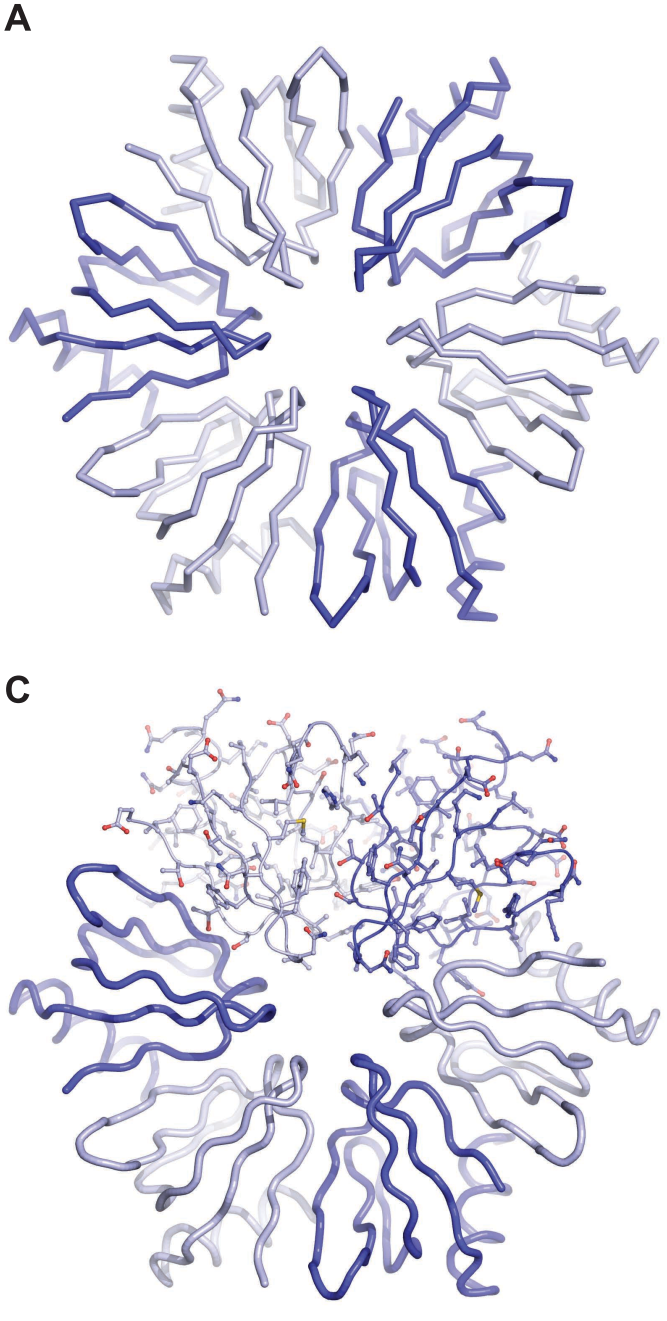

Figure S2. Different representation styles and their relative utility. *(cont'd)*

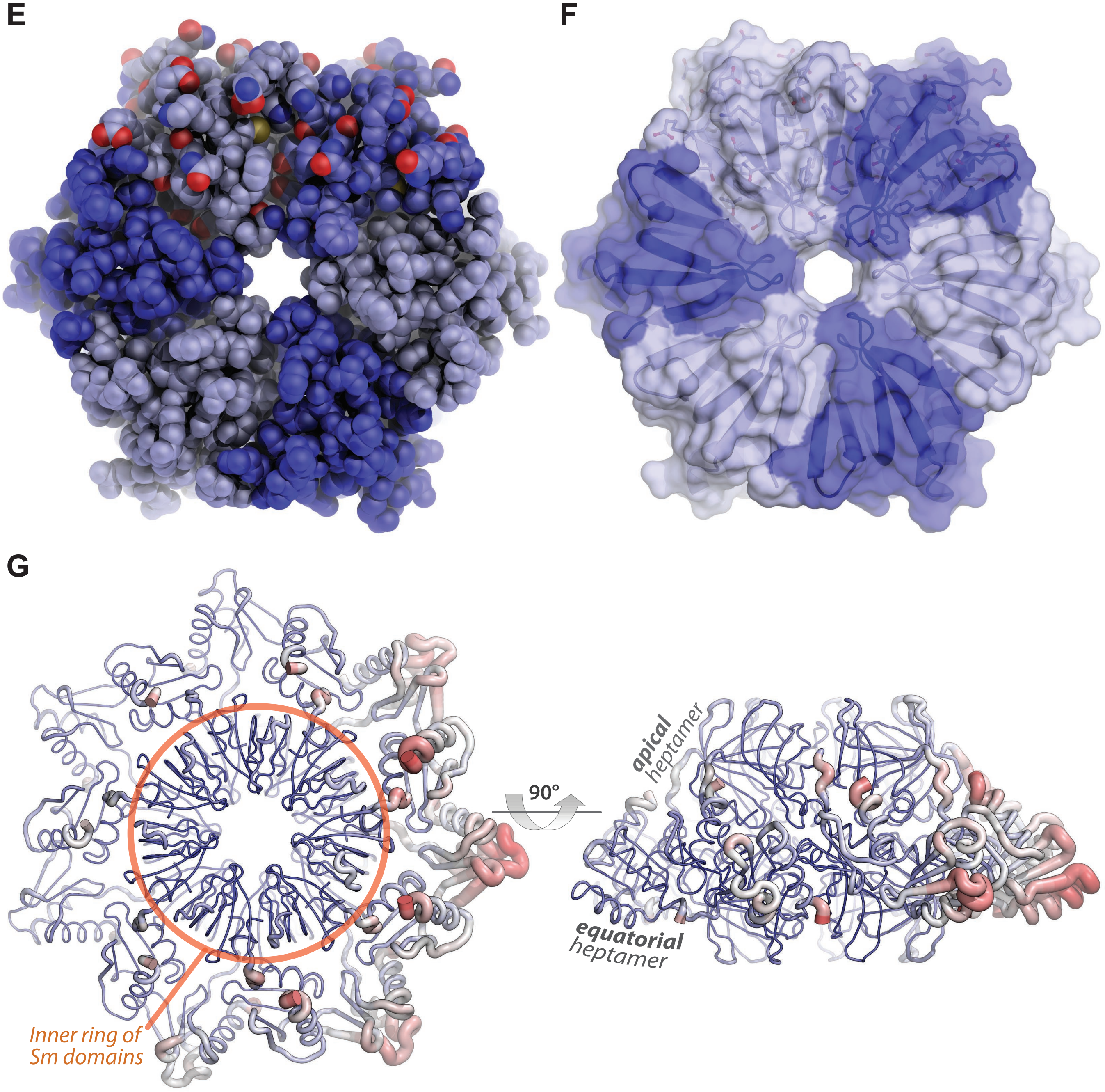

Supplement: Figure S2 — Different representation styles and their relative utility. (3.10 MB PDF) [file pcbi.1000918.s003.pdf]
