# Supplementary material for: An Introduction to Biomolecular Graphics
Source: PLoS Comput Biol. 2010 Aug 26;6(8):e1000918. doi: 10.1371/journal.pcbi.1000918 (PMC2928806; doi:10.1371/journal.pcbi.1000918)

Figure S3. A tetradecamer assembly: Overall architecture, bipartite domain organization, and Cd^{2+}-binding sites.

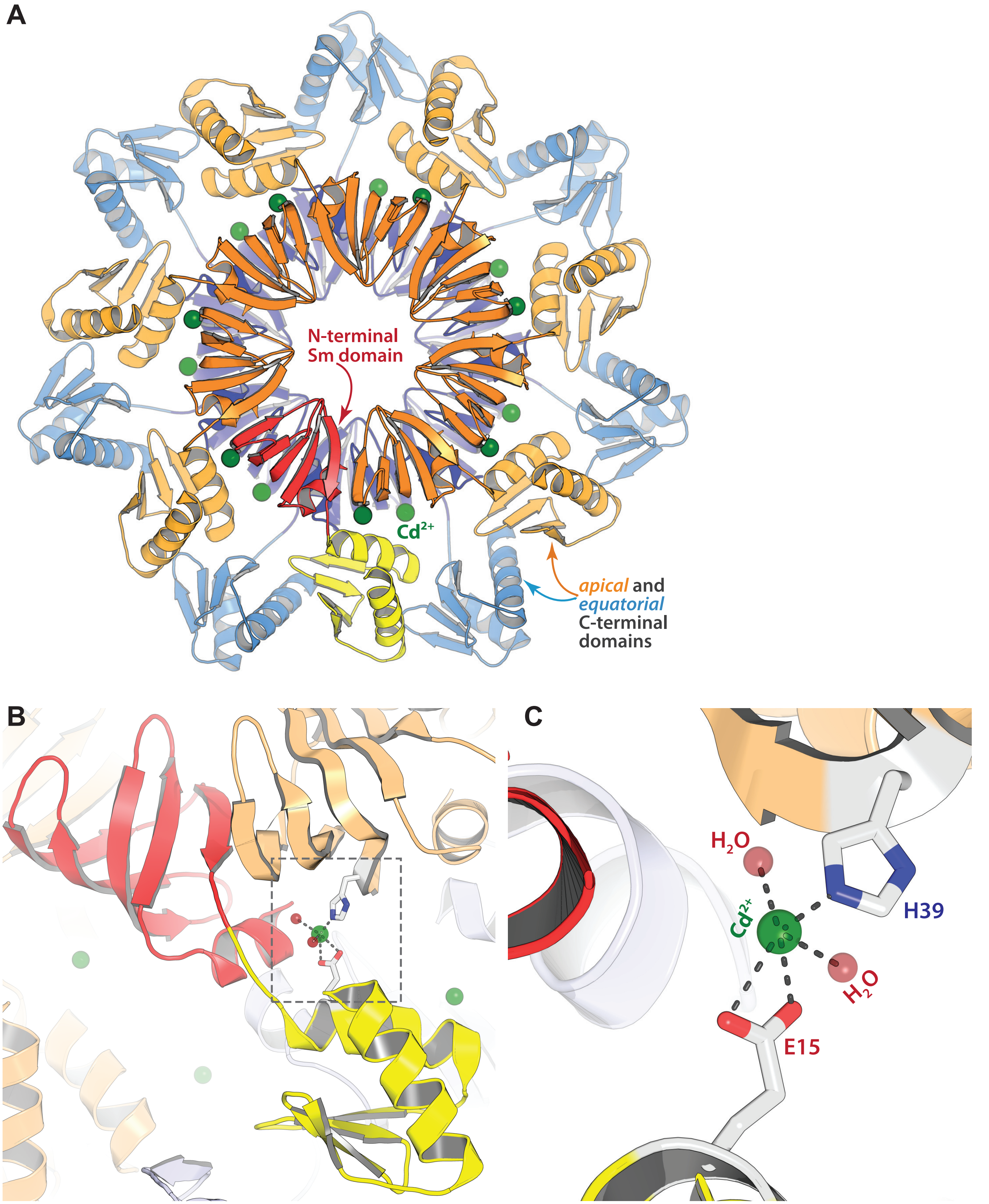

Supplement: Figure S3 — A tetradecamer assembly: Overall architecture, bipartite domain organization, and Cd2+-binding sites. (7.91 MB PDF) [file pcbi.1000918.s004.pdf]
