# Supplementary material for: An Introduction to Biomolecular Graphics
Source: PLoS Comput Biol. 2010 Aug 26;6(8):e1000918. doi: 10.1371/journal.pcbi.1000918 (PMC2928806; doi:10.1371/journal.pcbi.1000918)

Figure S4. Representations of volumetric data: Electron density and electrostatic potentials.

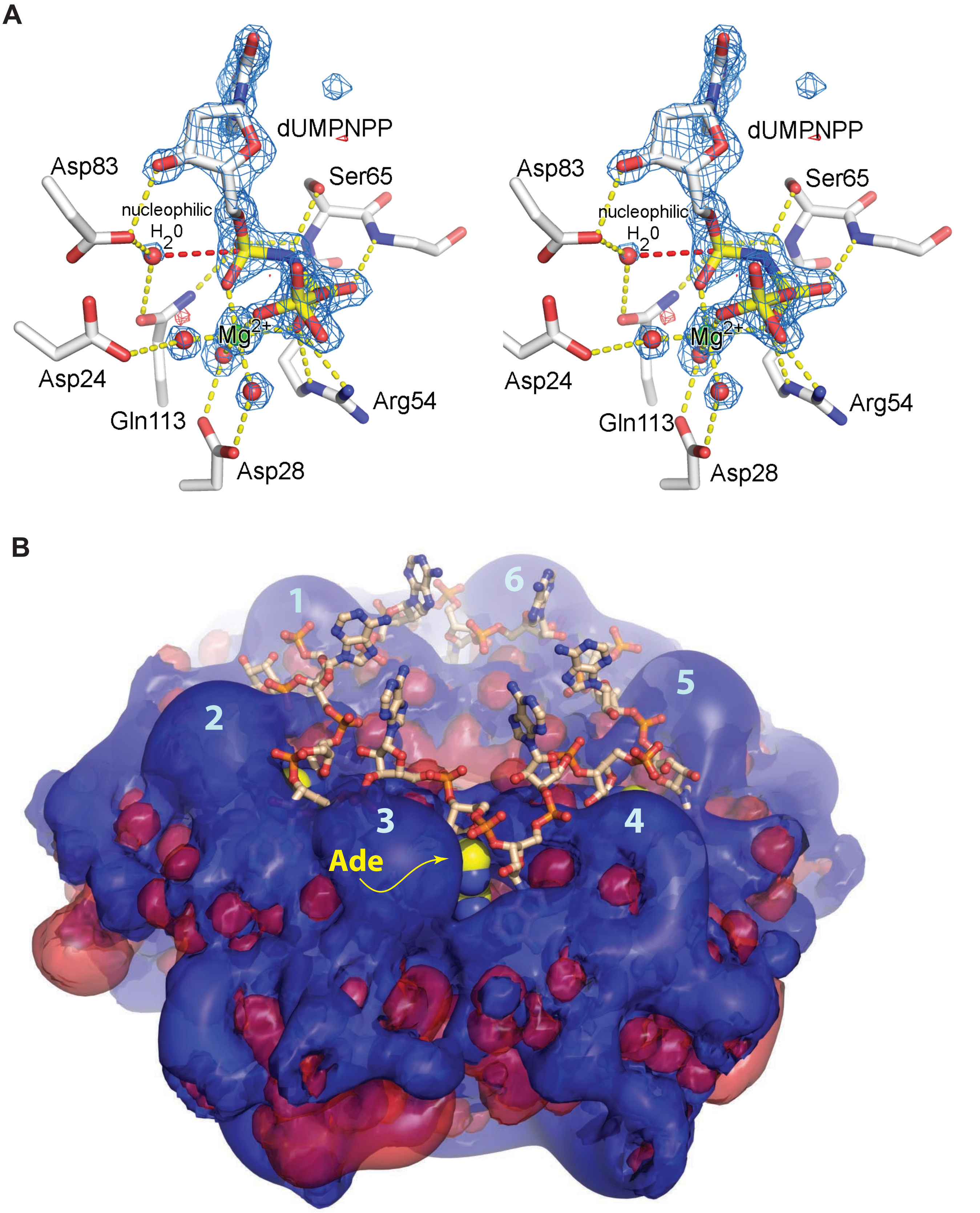

Figure S4. Representations of volumetric data: Electron density and electrostatic potentials.

C

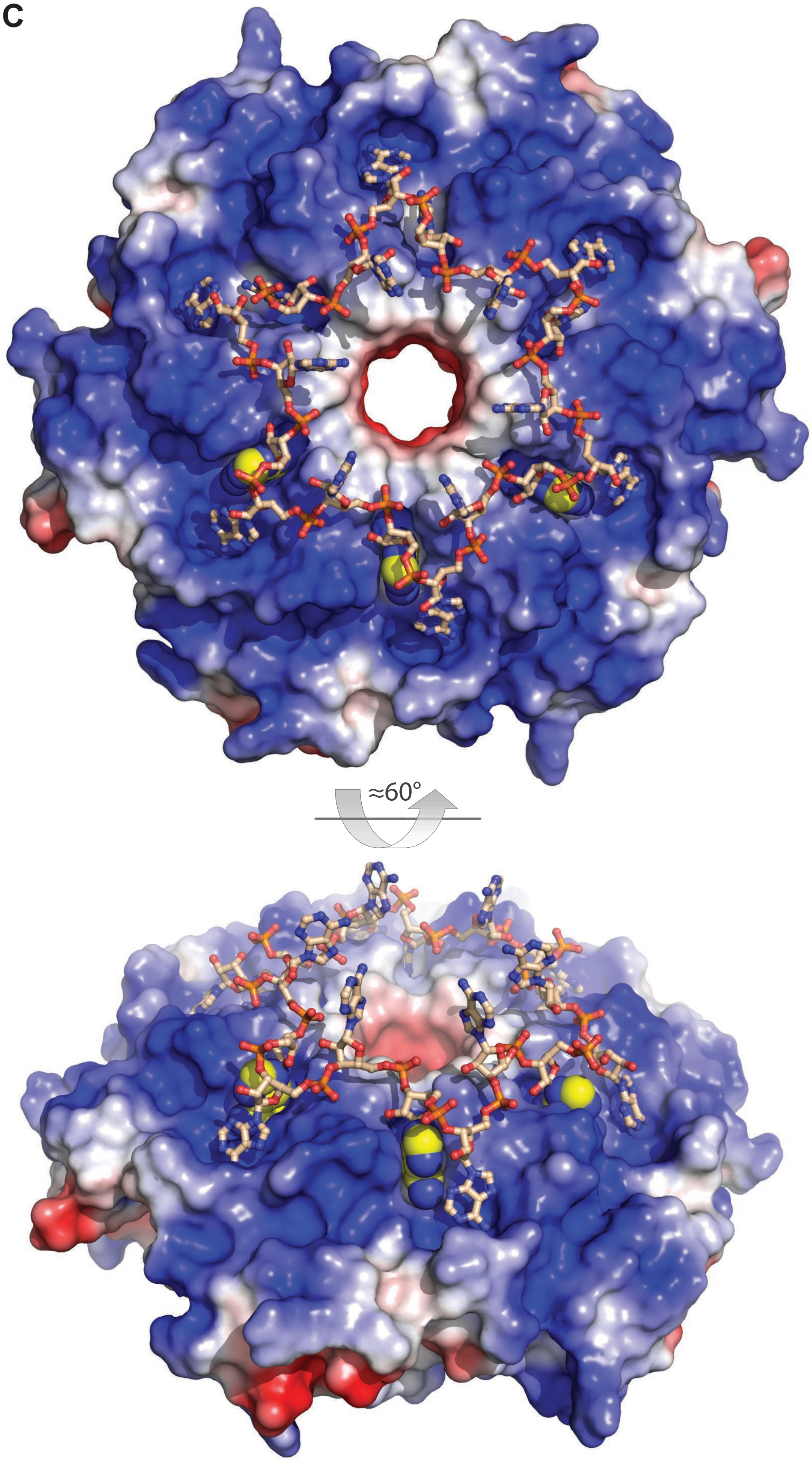

Figure S4. Representations of volumetric data: Electron density and electrostatic potentials. *(cont'd)*

D

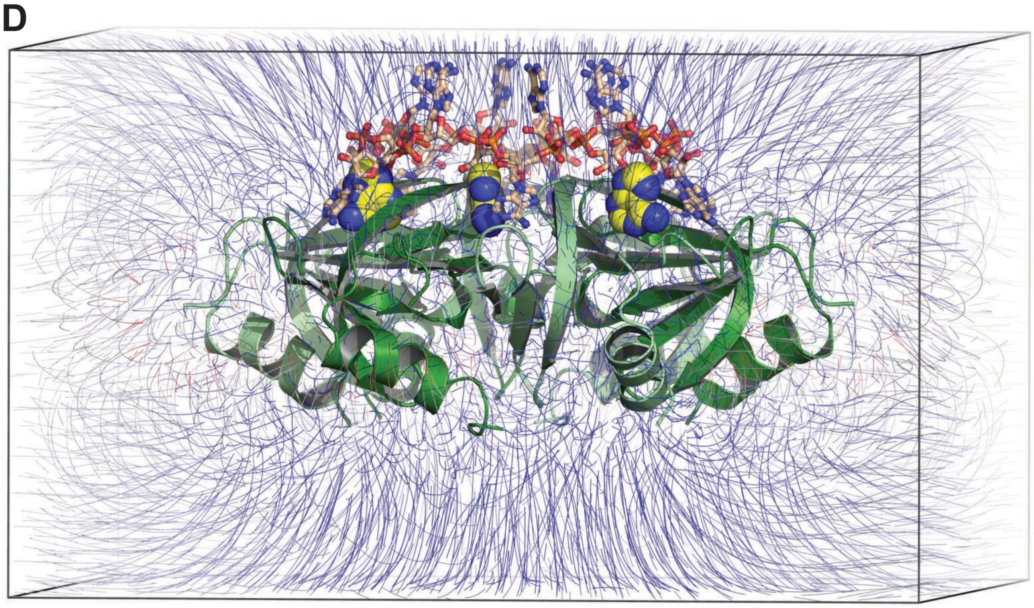

E

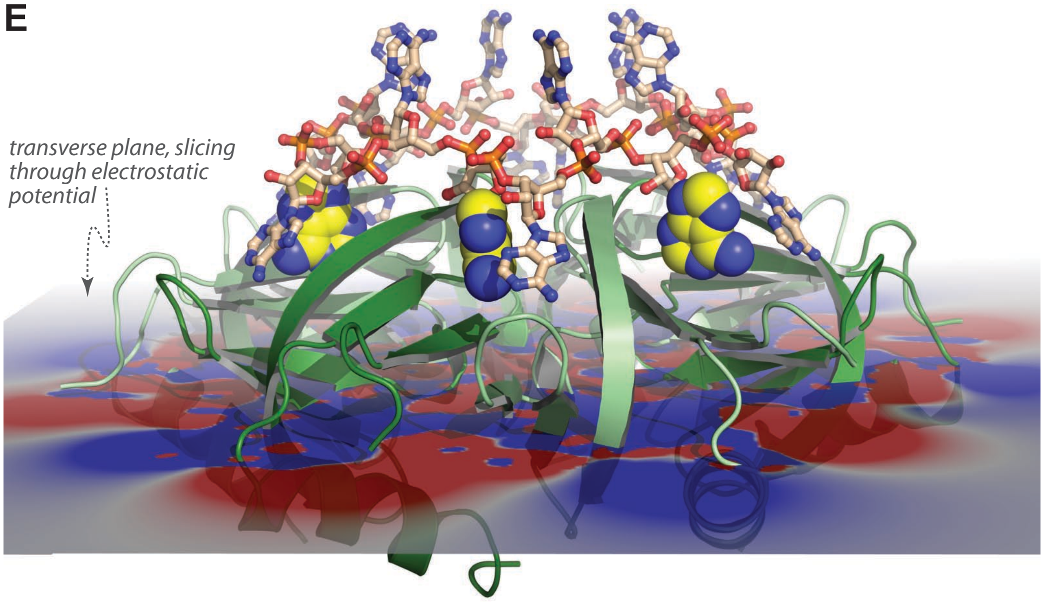

Supplement: Figure S4 — Representations of volumetric data: Electron density and electrostatic potentials. (3.29 MB PDF) [file pcbi.1000918.s005.pdf]
